# Compatibility of Distinct Label-Free Proteomic Workflows in Absolute Quantification of Proteins Linked to the Oocyte Quality in Human Follicular Fluid

**DOI:** 10.3390/ijms22147415

**Published:** 2021-07-10

**Authors:** Aleksandra E. Lewandowska, Anna Fel, Marcel Thiel, Paulina Czaplewska, Krzysztof Łukaszuk, Jacek R. Wiśniewski, Stanisław Ołdziej

**Affiliations:** 1 Intercollegiate Faculty of Biotechnology UG&MUG, University of Gdańsk, Abrahama 58, 80-307 Gdańsk, Poland; anna.fel@phdstud.ug.edu.pl (A.F.); marcel.thiel@ug.edu.pl (M.T.); paulina.czaplewska@ug.edu.pl (P.C.); 2 INVICTA Fertility and Reproductive Center, Polna 64, 81-740 Sopot, Poland; luka@gumed.edu.pl; 3 Department of Obstetrics and Gynecological Nursing, Faculty of Health Sciences, Medical University of Gdańsk, Dębinki 7, 80-211 Gdańsk, Poland; 4 Department of Proteomics and Signal Transduction, Max-Planck-Institute of Biochemistry, Am Klopferspitz 18, 82152 Martinsried, Germany; jwisniew@biochem.mpg.de

**Keywords:** LC-MS/MS, Total Protein Approach, SWATH-MS, human follicular fluid, proteome, oocyte quality control, oocyte maturity, blastocyst development

## Abstract

We present two separate label-free quantitative workflows based on different high-resolution mass spectrometers and LC setups, which are termed after the utilized instrument: Quad-Orbitrap (nano-LC) and Triple Quad-TOF (micro-LC) and their directed adaptation toward the analysis of human follicular fluid proteome. We identified about 1000 proteins in each distinct workflow using various sample preparation methods. With assistance of the Total Protein Approach, we were able to obtain absolute protein concentrations for each workflow. In a pilot study of twenty samples linked to diverse oocyte quality status from four donors, 455 and 215 proteins were quantified by the Quad-Orbitrap and Triple Quad-TOF workflows, respectively. The concentration values obtained from both workflows correlated to a significant degree. We found reasonable agreement of both workflows in protein fold changes between tested groups, resulting in unified lists of 20 and 22 proteins linked to oocyte maturity and blastocyst development, respectively. The Quad-Orbitrap workflow was best suited for an in-depth analysis without the need of extensive fractionation, especially of low abundant proteome, whereas the Triple Quad-TOF workflow allowed a more robust approach with a greater potential to increase in effectiveness with the growing number of analyzed samples after the initial effort of building a comprehensive spectral library.

## 1. Introduction

The accurate evaluation of the oocyte quality remains one of the main challenges in reproductive sciences. Although the proteomic analysis of a single oocyte cell has been recently accomplished [1], the oocyte cannot simultaneously be subjected to invasive analysis and maintain its conceptive potential. A consequence of those issues is a popular interest in the proximate environment of the developing oocyte, i.e., granulosa (GC) and cumulus cells or follicular fluid (FF) [2,3]. Human follicular fluid (hFF) from a single follicle is harvested along with the oocyte in the ovarian puncture procedure during in vitro fertilization (IVF) treatment. Therefore, it constitutes an easily obtainable material for the analysis of the processes occurring around the oocyte at the time of sample collection and has been appointed as a candidate for oocyte quality biomarker research [2,3]. Most of studies on hFF focused on utilizing diverse methodological strategies to characterize its proteome composition. Due to the proximity of hFF to human serum, its proteomic analysis faces the same obstacles related to high diversity in dynamic protein concentrations. The use of distinct sample preparation workflows including fractionation schemes facilitated the identification of the most comprehensive sets of hFF proteins containing from few hundreds to 2461 proteins (analysis of human small antral follicle fluid—hSAF by Pla et al.) [4,5,6,7,8,9,10]. Quantitative hFF studies often concern analysis of pooled samples (coming from multiple follicles from one or more donors) [11] or single dominant follicles of each donor [6,12], yet it is essential that the oocyte quality studies (as methods and techniques used) must focus on multiple hFF samples related to individual oocytes from single donors [10,13,14]. Investigation of samples related to individual oocytes increases the number of measurements that should be performed and not all techniques or methods utilized so far in hFF proteome studies are well suited for large-scale clinical studies due to the cost of analysis, time and labor burden, or other factors. In order to achieve an in-depth analysis of hFF proteomic landscape and to propose an optimal methodology for the identification of proteins related to individual oocyte quality in a large-scale clinical study (involving many samples), we employed diversified sample preparation schemes to develop optimized quantitative protocols utilizing two independent instrument setups including the following high-resolution mass spectrometers: Q Exactive HF-X coupled with nano-LC and TripleTOF 5600+ coupled with micro-LC. The Total Protein Approach (TPA) [15] was applied for data registered on both instruments to achieve absolute protein concentrations in label-free workflows. The data-independent acquisition (DIA) method, Sequential Window Acquisition of all Theoretical Mass Spectra (SWATH-MS) [16], was employed to facilitate the quantification on TripleTOF 5600+ in a DIA-TPA workflow [17] (further referred to as the Triple Quad-TOF workflow), whereas the data-dependent acquisition (DDA) mode on Q Exactive HF-X was utilized in a standard DDA-TPA workflow [15] (further referred to as the Quad-Orbitrap workflow). The SWATH-MS method utilized on a less sensitive instrument setup allowed us to quantify lower abundant proteins in unfractionated samples by paying a one-time resource cost of extensive library construction. We illustrated the analytical capacity of our approaches in a small-scale pilot study of 20 hFF samples from four donors and derived lists of proteins possibly linked to oocyte maturity and resulting blastocyst development status. As a result, we demonstrate the compatibility of both distinct workflows in protein quantification and propose a relatively high-throughput methodological strategy of analysis of hFF proteomic composition that has potential applicability to a large clinical study planned in the future.

## 2. Results

### 2.1. Experimental Design

In this study, we have developed and optimized two distinct quantitative workflows for the analysis of the hFF material: (i) The Quad-Orbitrap workflow utilizing the Q Exactive HF-X instrument coupled to nanoL and facilitated by the DDA-TPA data analysis on the MaxQuant database search results and (ii) the Triple Quad-TOF workflow with MS measurements on the TripleTOF 5600+ coupled to microLC analyzed in the DIA-TPA method using ProteinPilot as a database search software. We have tested several analytical strategies of hFF proteomic investigation to optimize both workflows. The scheme of sample preparation involved stages of protein fractionation, proteolytic digestion, and peptide fractionation. Every experiment combined different techniques, but did not necessarily include all stages. The sample preparation scheme and instrument used in each experiment are listed in Table 1 (see Table S1 for more details).

The Quad-Orbitrap workflow was aimed at the highest proteomic coverage with an inclusive quantitative analysis. Therefore, we analyzed the MED-FASP samples on the higher resolution-instrument in longer nanoLC gradients; a procedure which consumed the most time and resources in each single analysis. On the other hand, we designed the Triple Quad-TOF workflow to provide a higher throughput while maintaining a reasonable depth of proteomic analysis. For that reason, we performed shorter MS analysis of samples after only one digestion in the FASP procedure. However, using the advantage of the SWATH-MS methodology, we performed multiple fractionation experiments and utilized the results in the spectral library creation. In that manner, we went through the one-time resource cost to increase the quantification capability of the unfractionated clinical samples.

Due to instrument availability, more extensive investigation, including a number of fractionation experiments, was conducted on TripleTOF 5600+. Briefly, we have tested four methods of protein digestion: in solution digestion by trypsin; FASP by trypsin, MED-FASP with consecutive digestions by LysC, trypsin, and chymotrypsin; and MED-FASP with consecutive digestions by trypsin and chymotrypsin. Two of those, in solution digestion and MED-FASP with three enzymes, were employed in the analyses on both instruments and the other two were only used for samples analyzed on TripleTOF 5600+. We utilized three fractionation techniques. The first technique of protein fractionation applied in the study is ultrafiltration. We obtained two fractions after ultrafiltration on 10 kDa cutoff membrane Amicon filters: HMWF containing proteins as the retentate and LMWF containing endogenous peptides as the filtrate. HMWF was further digested, while LMWF was prepared for MS measurements without proteolytic digestion to identify peptides resulting from physiological protein breakdown in hFF. Next applied protein fractionation method was immunodepletion of high abundant proteins by the use of MARS Hu-14 cartridge. We analyzed both the depleted protein fraction enriched in low abundant proteins (MF) and the resulting high abundant proteins fraction (MR). Lastly, the experiments involving high pH RP-HPLC fractionation of peptides were conducted only on the TripleTOF 5600+ instrument to increase the proteomic coverage of the subsequently utilized SWATH-MS spectral library.

### 2.2. The Quad-Orbitrap Workflow

#### 2.2.1. Workflow Optimization

**Figure 1:**
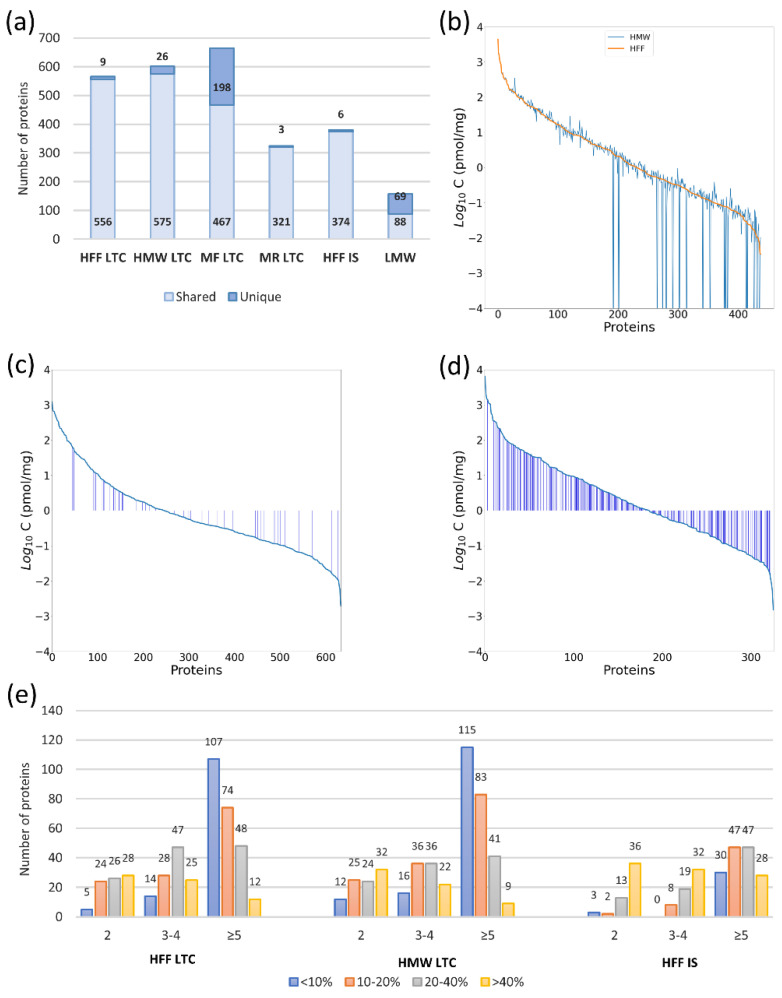
The effect of sample preparation on the identification and quantification of the result in the Quad-Orbitrap workflow. Sample names refer to abbreviations listed in Table 1 without the instrument designation (QE). (**a**) Numbers of proteins identified in all single experiments segregated into proteins found in multiple experiments (shared) and identified only in one experiment (unique). (**b**) Difference in protein concentration analyzed in the unfractionated (HFF) and fractionated by ultrafiltration (HMW) samples. Proteins are arranged in a decreasing mean concentration order in the unfractionated sample. Zero values in HMW experiment were implemented as 10^−6^ in order to present them in the chart. (**c**,**d**) Quantification of proteins after immunodepletion: low abundant protein fraction (**c**) and high abundant protein fraction (**d**). In each chart, proteins are arranged in a decreasing order of concentration. Blue bars represent proteins present in lower concentration than in the unfractionated sample. (**e**) Evaluation of protein quantification of samples: unfractionated sample digested by MED-FASP, unfractionated sample digested in solution, and sample fractionated by ultrafiltration digested by MED-FASP. Bars represent numbers of proteins quantified by a given number of peptides (grouped into columns) and at a given coefficient of variation (CV) value (showed in different colors).

We identified 942 proteins in all experiments conducted on the Q Exactive HF-X instrument using two methods of sample fractionation (immunodepletion and ultrafiltration, see Figure 1a–e) and two methods of sample digestion (MED-FASP and in solution digestion, see Figure 1a,e). The results for single experiments are listed in Table S2 and their summaries are shown in Figure 1a.

Protein digestion by MED-FASP resulted in a higher yield of identified and quantified proteins in comparison to in solution digestion: 565 and 438 proteins, respectively (1.49-fold and 1.65-fold more). Moreover, the quality of quantification was better in MED-FASP digested samples, which is especially evident in the numbers of proteins quantified by five and more peptides and their much lower CV values. Therefore, MED-FASP was employed as the method of choice in subsequent experiments.

An additional 36 identifications were achieved in HMWF after fractionation by ultrafiltration in comparison to unfractionated samples and 26 proteins were detected exclusively in HMWF. Moreover, a substantial part of LMWF proteins were found only in this fraction (44% unique identifications). Numbers of proteins quantified in HMW and unfractionated samples were very similar and so was the quality of quantification. However, the differences in absolute concentrations between those samples were apparent (see Figure 1b). With lowering concentration, the concentration deviation becomes higher, yet even medium abundant proteins display alterations in their concentration.

The other applied fractionation technique was immunodepletion using the MARS-14 cartridge. A lot more proteins were captured by the cartridge than the anticipated 14 HAPs (high abundant proteins), resulting in 324 identifications. However, HAP fraction generally contained fewer identified proteins than the unfractionated sample. Conversely, the immunodepleted fraction was the richest in identifications among all experiments with 665 total and 198 uniquely identified proteins. Figure 1c,d depict the changes in concentrations of proteins quantified by the Quad-Orbitrap workflow in HAPs and immunodepleted fractions, respectively, in comparison to the unfractionated sample. Most proteins in the HAPs fraction are present at lower concentrations than in the unfractionated sample (proteins designated as blue bars in Figure 1c,d); however, general protein concentrations are mostly relatively high. On the other hand, a very small fraction of proteins in the immunodepleted fraction are present at lower concentrations than in the unfractionated sample, yet most proteins are present at relatively low concentrations and obviously more proteins in this fraction could be quantified.

#### 2.2.2. Functional Enrichment Analysis on Interaction Networks Created in STRING Database

We divided the list of 438 proteins quantified by the Quad-Orbitrap workflow in an unfractionated hFF sample (see Table S3) ranked by their absolute concentration according to their abundance into three groups: (i) high abundant proteins (HAPs, 95% of total quantified proteins’ mass; 72 most abundant proteins), (ii) medium abundant proteins (MAPs, 4.5% of total quantified proteins’ mass; 102 proteins subsequent in concentration), and (iii) low abundant proteins (LAPs, 0.5% of total quantified proteins’ mass; 264 low abundant proteins). The most abundant protein, which was serum albumin, was present at 4442.45 pmol/mg (30.82% of all protein content) and the least abundant protein in HAPs group was immunoglobulin lambda-like polypeptide 5, which was present at 51.76 pmol/mg (0.12%). Furthermore, top 20 proteins added to 75.70%, whereas top 10 proteins constituted as much as 64.93% of all protein content. One hundred and two lower abundant proteins were grouped as MAPs, which comprised approximately 4.5% of total quantified proteins’ content. The first protein in this group was immunoglobulin lambda variable 1–47 (92.4 pmol/mg, 0.11%) and the last protein was immunoglobulin heavy variable 4–4 (9.33 pmol/mg, 0.01%). The least abundant proteins numbering 264 were classified as LAPs and comprised approximately 0.59% of the total quantified proteins’ content. Moreover, we also included 309 proteins quantified by the Quad-Orbitrap workflow only after immunodepletion with the use of MARS-14 kit in our functional analysis to present a group of proteins masked by the presence of HAPs.

We created interaction networks for each protein group using the STRING database [18] and focused on the functional enrichment analysis for three categories: biological process (GO), molecular function (GO), and Reactome [19] pathways (Table S4). The reason for omitting other categories were multiple instances of repeating terms, the most notable were the following: extracellular region in cellular component (GO), signal or secreted in UniProt keywords, and coagulation cascade in KEGG [20] pathways (excluding LAPs group and group of proteins quantified only after immunodepletion with ECM-receptor interaction and pentose phosphate pathway as the top term, respectively). HAPs are mostly associated with platelet degranulation, protein activation cascade, enzyme (notably endopeptidase) inhibitor and regulator activity, and regulation of IGF transport and uptake by IGFBPs. However, the fact that not all protein identifiers were imported into the STRING database must be noted, as multiple immunoglobulin-related entries were discarded at that point. MAPs are also linked to the protein activation cascade; however, more focus is placed on complementing cascade regulation and the immune system. Interestingly, their molecular function is related to peptidase activity and its regulation. LAPs and proteins quantified only after immunodepletion were similar in terms of results, which included the following terms: regulated exocytosis, biological adhesion, protein and other molecule binding, and immune system.

As a next step, we also created interaction networks in the described manner for proteins identified in LMWF by the Quad-Orbitrap workflow: (i) all 157 identified proteins and (ii) 69 proteins identified exclusively in LMWF. In the case of all LMWF proteins (Figure S1a), many terms overlap with previous HMWF analysis: platelet degranulation, endopeptidase inhibitor activity, peptidase regulator activity, or hemostasis (Table S5). Moreover, even though most proteins were also assigned to extracellular region, we colored the resulting network according to different protein localization due to the formation of visible interaction clusters in the network. These clusters are mostly related to the nucleosome, ribonucleoprotein complex, and extracellular region and/or vesicle. Differences in assigned terms worth mentioning are the negative regulation of biological process, transport, and developmental biology. The network of proteins exclusive to LMWF consists mostly of proteins localized in nucleosome and ribonucleoprotein complex (Figure S1b). Therefore, most of the enriched functional terms in this network are related to translation and accompanying processes.

### 2.3. The Triple Quad-TOF Workflow

**Figure 2:**
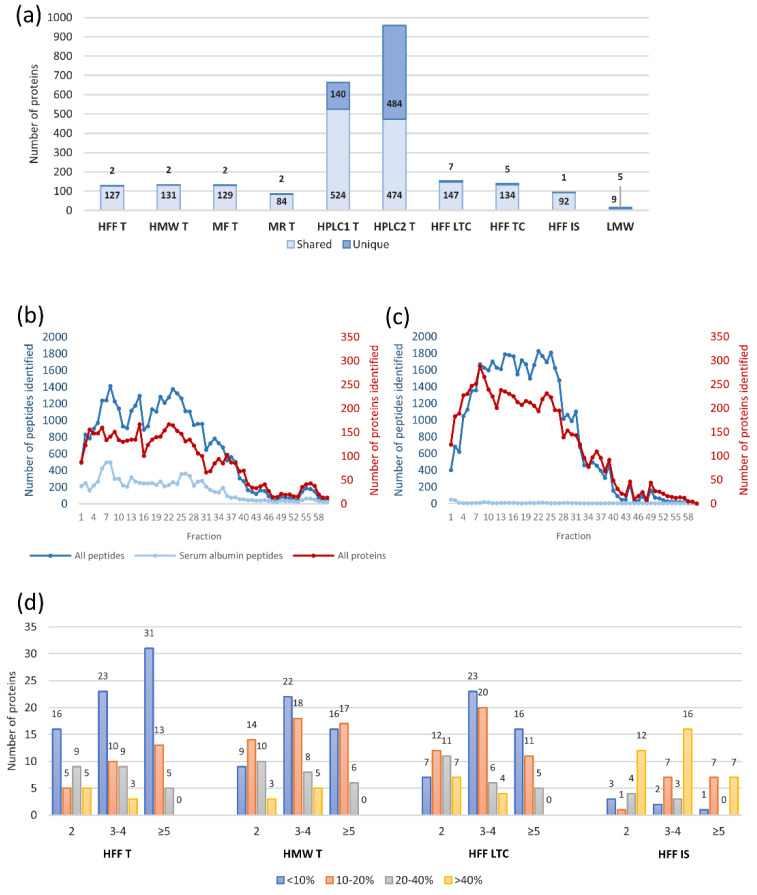
The effect of sample preparation on the identification and quantification result in the Triple Quad-TOF workflow. Sample names refer to abbreviations listed in Table 1 without the instrument designation (3TOF) (**a**) Numbers of proteins identified in all single experiments segregated into proteins found in multiple experiments (shared) and identified only in one experiment (unique). (**b**,**c**) Numbers of proteins (red), all peptides (dark blue), and serum albumin peptides (light blue) in each fraction in high pH RP-HPLC experiments: without prior protein fractionation—HPLC1; (**b**) and after immunodepletion of high abundant proteins—HPLC2 (**c**). (**d**) Evaluation of protein quantification of samples: unfractionated samples digested by MED-FASP, FASP, or in solution and sample fractionated by ultrafiltration digested by FASP. Bars represent numbers of proteins quantified by a given number of peptides (grouped into columns) and at a given coefficient of variation (CV) value (showed in different colors).

Initially, we tested the efficiency of the Triple Quad-TOF workflow in 1 h and 30 min LC gradients using sample preparation methods analogous to the Quad-Orbitrap workflow (see Section 2.2.1). In these experiments, we identified approximately 5 to 20% less proteins using shorter gradients, depending loosely on the complexity of the sample (e.g., 5% less in case of unfractionated sample digested in solution and 20% less in immunodepleted fraction digested by FASP). The 30 min LC gradient was selected for the study due to the relatively small decrease in identifications as compared to the substantial advantage of shorter analysis time. Using these short LC-gradients, we identified 259 proteins in total (results presented on Figure 2a–d).

In order to test if we could enhance these results, we conducted high pH RP-HPLC peptide fractionation. Thus, we obtained 1151 identifications in those experiments alone at the cost of additional work labor and instrument analysis time. Only 31 proteins identified in other experiments were not detected after this method of fractionation, which taken together resulted in a total of 1182 identifications. The results for single experiments are listed in Table S6 and their summaries are shown in Figure 2a.

MED-FASP was more effective than in solution digestion in terms of identification and quantification of proteins to an even higher extent than in the case of the Quad-Orbitrap workflow resulting in 154 and 122 proteins, respectively (1.66-fold and 3.49-fold more). Additionally, we tested the FASP digestion with a single enzyme (trypsin) to simplify the sample preparation protocol for the purpose of the SWATH-MS quantification (see Table S7). Here, we obtained a lower number of identifications than in the case of MED-FASP (25 proteins less); however, the quantification parameters have improved. We were able to quantify only seven more proteins, yet the total number of proteins quantified at CV < 10% rose from 46 to 70. We examined MED-FASP digestion with two enzymes only in terms of identified proteins and obtained a number between identifications for FASP digested samples and MED-FASP digested samples, which is as expected. Due to much-improved quantification results, we applied the FASP digestion with trypsin in preparation of other samples in the Triple Quad-TOF workflow. Moreover, we examined the quantification capabilities of this method also in the 1 h LC gradient and observed a 10% increase in quantified proteins using basic libraries without the high pH RP-HPLC measurements (126 proteins in 1 h as compared to 113 proteins in 30 min).

Fractionation by ultrafiltration resulted in a similar number of identified and quantified proteins in the HMW fraction as in the unfractionated sample (with 16 new identifications in HMW in respect to HFF). However, we observed a substantial decrease in lower CV values (<10%) for HMW samples. The analysis of LMW fraction was much less comprehensive in comparison to the Quad-Orbitrap workflow, yet we also managed to identify a few unique proteins. Immunodepletion also produced similar identification results, however, as much as 69 proteins identified in immunodepleted fraction constituted identifications that were not found in the unfractionated sample. The HAPs fraction was characterized by the smallest number of identified proteins.

To obtain a more comprehensive view of hFF proteome with the use of TripleTOF 5600+, we employed the high pH RP-HPLC fractionation of peptides and additionally performed an experiment, where we coupled it with prior protein immunodepletion. The use of this peptide fractionation method allowed us to achieve a similar or slightly higher number of protein identifications as with the use of Q Exactive HF-X when applied as a standalone technique (664 identifications, 140 unique) and to significantly exceed it in combination with immunodepletion (958 identifications, 484 unique). Nonetheless, this gain in identifications comes at a price of a significant instrument time increase as each experiment consisted of 60 separately analyzed fractions. Figure 2b,c demonstrates chromatogram-like charts of numbers of proteins, peptides, and peptides derived from albumin in each analyzed fraction. Both experiments show that proteins were mainly identified in the first 40 fractions and in especially high numbers in the first 25 fractions. Peptides derived from albumin were present in each fraction in the first experiment, possibly overshadowing other identifications, which could be detected after albumin depletion in the combined experiment.

### 2.4. Literature Comparison to Proteomic Studies of hFF and Related Biological Materials

In the conducted literature comparison, we focused on a few of the most comprehensive proteomic studies of human follicular fluid up to date: Zamah et al., 2015 (742 reported proteins) [6]; Bianchi et al., 2016 (617 reported proteins) [5]; Oh et al., 2017 (1079 reported proteins) [7]; Poulsen et al., 2019 (400 reported proteins) [4]; Zhang et al., 2019 (1153 reported proteins) [9]; and Pla et al., 2020 (2461 reported proteins) [10]. Furthermore, we compared the set of proteins identified in this study to the reported proteomes of biological materials in the proximate physiological environment, i.e., human plasma (Plasma Proteome Database—10,546 reported proteins) [21]; human oocyte (Virant-Klun et al., 2016—2154 reported proteins) [1]; and human granulosa cell (Bagnjuk et al., 2019—3642 reported proteins) [22]. The detailed comparison for each protein is presented in Table S8 and the general overview with cumulative data is presented in Table 2.

As expected, most of the proteins identified in this study were also reported in human plasma: about 82% of all sets with the exception of LMWF-specific proteins. The next most overlapping proteome is the granulosa cell (about 45–50%) with a slight increase in overlap in LMWF. Lastly, about 22–27% of proteins were reported in human oocyte, 22 of which were described as oocyte-specific; most of those were identified in HMWF. In comparison to hFF proteomic studies mentioned above, from almost 28% (Poulsen et al. 2019 to Triple Quad-TOF-identified proteins) to about 88% (Pla et al. 2020 to Quad-Orbitrap-identified proteins) of identifications overlapped. The numbers of the same identifications relied mostly on the total number of proteins identified in the study. The overwhelming majority of proteins reported in other hFF studies were found in the HMWF with a total of 42 proteins detected only in the LMWF. Of the proteins studied, 199 and 63 proteins identified in our study were not reported in the analyzed hFF publications and in any of the referred to sources, respectively.

### 2.5. Compatibility of the Quad-Orbitrap and Triple Quad-TOF Workflows

#### 2.5.1. Quantification Capability in Pooled Material

**Figure 3:**
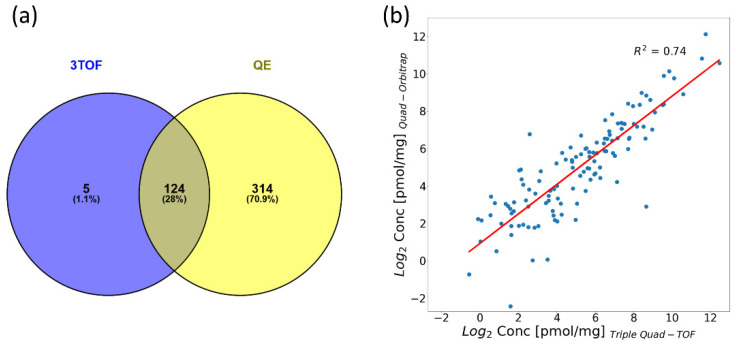
General comparison of compatibility of quantification capabilities of tested workflows. (**a**) Venn diagram illustrating numbers of quantified proteins by the Triple Quad-TOF (3TOF) and Quad-Orbitrap (QE) workflows. (**b**) Scatterplot of log2-transformed median concentration values from the Triple Quad-TOF and Quad-Orbitrap workflows.

We were able to quantify 129 and 438 proteins in an unfractionated hFF sample using the Triple Quad-TOF and Quad-Orbitrap workflows, respectively (Figure 3a, Tables S3 and S7). Five proteins were quantified exclusively by Triple Quad-TOF workflow. The majority of all proteins were quantified only with the use of the Quad-Orbitrap workflow—314 proteins. We obtained the Pearson correlation coefficient value of 0.86 for median concentrations of 124 proteins measured by both workflows and presented the scatterplot demonstrating their compatibility in Figure 3b.

#### 2.5.2. Pilot Study on Clinical Samples

We set up a small-scale pilot study to further examine quantitative capabilities of the investigated methods and their effectiveness in elucidation of biological context in the clinical setup. Twenty hFF samples from individual follicles of four patients were included in the study (see Table S9). Selected samples differed in the maturity of retrieved oocyte and the resulting blastocyst status was obtained after embryo culture of fertilized mature oocytes. Thus, it allowed us to investigate differences in protein concentrations associated with oocyte quality along with differences stemmed from individual characteristics of patients.

Using both methodologies, we were able to quantify 471 proteins in total and 199 shared proteins by both methods (455 by the Quad-Orbitrap workflow and 215 by Triple Quad-TOF workflows, see Tables S10 and S11). We checked the consistency of obtained data for both methods by multiscatter plots (see Figures S2 and S3). P1F2 and P2F5 samples showed the lowest correlation with other samples, however, the P1F2 sample had such characteristics only in the case of the Triple Quad-TOF measurements which points to the possibility of error occurrence during the data acquisition of this sample. To compare the compatibility of the Quad-Orbitrap workflow and Triple Quad-TOF workflows in a general sense, we determined Pearson correlation values of concentrations: (i) between all clinical samples (Table S12) and (ii) in each sample resulting from both methods for each protein (Table S13). All samples presented inter-workflow correlation coefficient values higher than 0.75, excluding the P1F2 sample in which the correlation coefficient value was lower than 0.7. In the whole set of samples, 24 proteins displayed correlation coefficient values higher than 0.7, while in the filtered set of 19 clinical samples (P1F2 sample was excluded), 38 proteins displayed correlation coefficient values higher than 0.7 (Figure S4). Due to this, we decided to eliminate the P1F2 sample from further analysis as a possible outlier. Moreover, the higher Pearson correlation value did not correspond to the overall protein abundance (see Table S13). For instance, serum albumin, which was present at the highest concentration in the analyzed material, demonstrated the value of 0.47, whereas C4b-binding protein alpha chain (less than 0.04% of mean pool protein content) displayed 0.9.

In order to discern proteome differences related to oocyte quality from individual characteristics of each patient, we employed a two-way ANOVA with one factor grouping of all individual samples from one patient (further referred to as the patient factor) and the second factor grouping according to the estimate of oocyte quality as shown in Table S9. The results of all conducted tests are listed in Table S14. As expected, proteome differences between patients exceeded characteristics associated with the assessed oocyte quality. At 5% FDR, almost 48% of all proteins measured by any method were associated with patient factor in comparison with the retrieved oocyte quality and about 45% in comparison with the blastocyst status, while at 1% FDR the proteins’ concentration changes were statistically significant for about one third of all proteins. Out of 199 proteins analyzed by both methods, 139 proteins in total with 34 detected in each comparison and a total of 98 proteins and 20 detected in each comparison were linked to the patient factor at 5% and 1% FDR, respectively (see Figure S5). We did not further explore the biological significance of the established inter-patient differences due to the limited number of patients included in the study.

Instead, we focused on the proteomic signatures related to oocyte quality. Two separate ANOVA tests were conducted: The first testing the group of mature oocyte versus immature oocyte outcomes (14 and 5 samples, respectively) and the second testing the group of fertilized mature oocytes that reached blastocyst stage versus those that were arrested in development before reaching blastocyst stage (8 and 6 samples, respectively; see Table S9). The numbers of proteins relevant for oocyte maturity status for both methods at 5% FDR were 49 and 10 for the Quad-Orbitrap workflow and Triple Quad-TOF workflows, respectively. Similarly, the numbers of proteins relevant for blastocyst status were 45 and 7, respectively. Out of those proteins, all Triple Quad-TOF-appointed quality-related proteins were also quantified by the Quad-Orbitrap workflow, whereas 23 out of 49 oocyte maturity-related proteins significant from the Quad-Orbitrap workflow and 30 out of 45 blastocyst-related proteins were quantified by the Triple Quad-TOF workflow. In order to illustrate the general results of statistical analysis, we created interaction networks for each group of statistically significant proteins resulting from one of the four comparisons (see Figure S6). Most of the designated proteins displayed only minimal concentration differences between tested groups. Eight proteins related to oocyte maturity in the Quad-Orbitrap experiments exceeded 2-fold concentration changes and all those proteins were not quantified in the Triple Quad-TOF analysis. Conversely, there were three such proteins detected in the Triple Quad-TOF analysis, including the only protein appointed to be significant by both methods, which is the hepatocyte growth factor-like protein. However, Triple Quad-TOF analysis displayed a decrease in the concentration of this protein in the test group (0.39), whereas the Quad-Orbitrap workflow pointed to a slight increase (1.09). The number of proteins related to blastocyst status with a fold change higher than two was also eight in the case of the Quad-Orbitrap quantification and two of those quantified in Triple Quad-TOF experiments, while there was only one such protein significant from the Triple Quad-TOF workflow. Again, only one protein, which was carboxypeptidase B2, was designated as statistically significant by both methods and was present at decreased concentrations in the developed blastocyst group.

**Figure 4:**
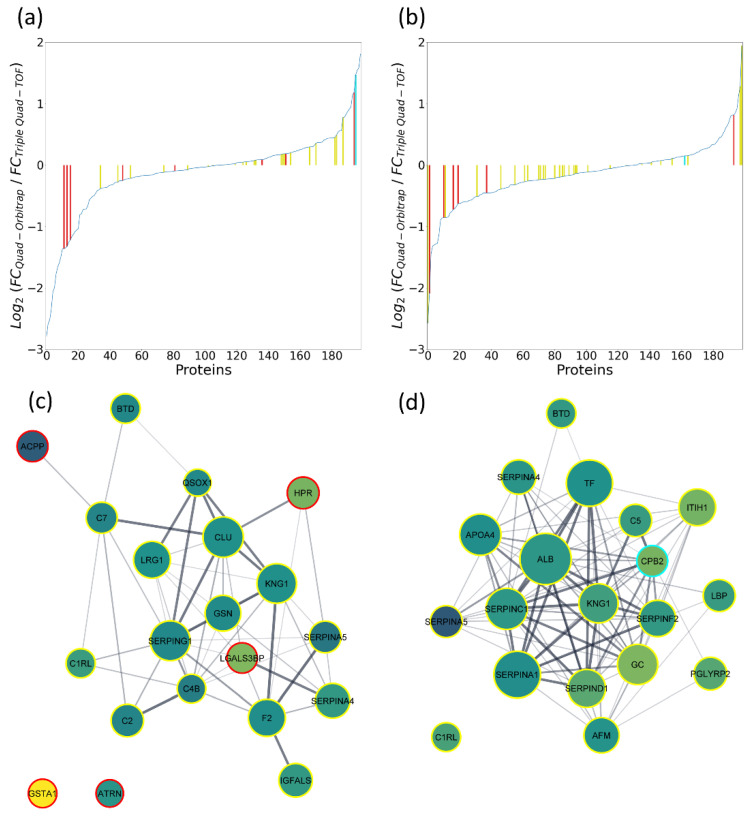
Proteins determined as statistically significant by two-factor ANOVA for the factor relating to (**a**,**c**) oocyte maturity or (**b**,**d**) blastocyst development status in both quantitative methods. (**a**,**b**) The ratio of fold change values of the Quad-Orbitrap workflow to Triple Quad-TOF workflow for each protein (log2). Statistically significant proteins are shown in colors (yellow—Quad-Orbitrap; red—Triple Quad-TOF; and both workflows—cyan). (**c**,**d**) Interaction networks for statistically significant proteins with less than 20%-fold change ratio difference between quantitative methods. Node edge color designates the quantitative method used to establish statistical significance (yellow—Quad-Orbitrap; red—Triple Quad-TOF; and both workflows—cyan). Fill color relates to the mean fold change of both methods, from 0.5 and below (yellow) through 1 (aquamarine) to 2 and above (purple). Node size represents mean log10 median abundance TPA concentrations of both methods in the test group (either mature oocyte or developed blastocyst).

In order to evaluate the relevance of the obtained lists of statistically significant proteins and to further compare quantification concurrence of both methods, we calculated ratios of median fold changes resulting from each comparison. Figure 4a, b presents charts showing differences for all 199 proteins quantified in both methods. This examination revealed the considerable agreement of quantification. For instance, less than one third of proteins were quantified with a fold change difference higher than 0.3, while more than one third of proteins displayed a fold change difference lower than 0.1 in both comparisons. Proteins determined as statistically significant by the Quad-Orbitrap workflow tended to cluster towards lesser differences in fold change ratio values in both comparisons. Proteins determined as statistically significant by the Triple Quad-TOF workflow displayed similar tendencies in the case of oocyte maturity comparison. On the other hand, most of the statistically significant proteins appointed by the Triple Quad-TOF workflow in the blastocyst status comparison have been measured with high discrepancies, except for a single protein, which was carboxypeptidase B2.

**Figure 5:**
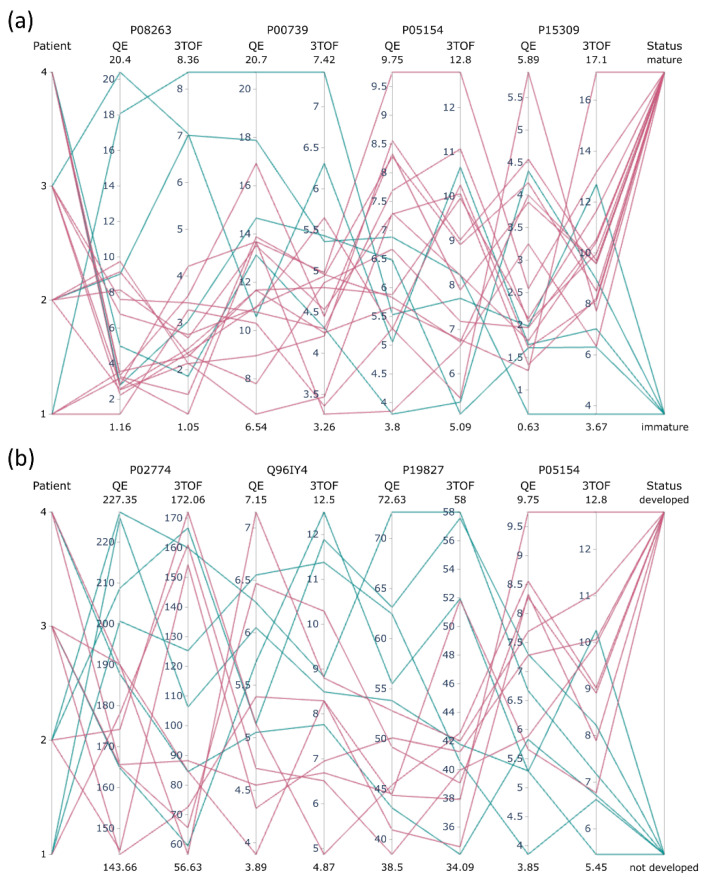
Median sample concentrations of proteins related to (**a**) oocyte maturity or (**b**) blastocyst development status with the highest fold changes measured by both workflows presented in parallel coordinates charts. Protein concentrations are scaled separately on each axis. Red and blue lines mark samples with the positive outcome (mature oocyte or developed blastocyst) and samples with the negative outcome (immature oocyte or not developed blastocyst), respectively.

We filtered the lists of proteins determined for each comparison to those presenting 0.2-fold or lower-fold change ratio differences between the methods and presented them as potential markers related to oocyte quality (see Table S15). As a result, we obtained 20 and 22 proteins related to oocyte and blastocyst status, respectively. In Table S15, we included literature reports showing connection of listed protein appearance and/or concentration in hFF with oocyte quality [4,6,10,11,12,13,14,23,24,25,26,27,28]. We constructed interaction networks for proteins associated with oocyte maturity (Figure 4c) and blastocyst status (Figure 4d). Proteins with extreme changes of concentrations were filtered out entirely, either due to no quantification in another method or high inconsistencies. Proteins related to oocyte maturity with the highest fold changes were glutathione S-transferase A1 and prostatic acid phosphatase (0.35 and 1.37 mean median fold change, respectively). Both of those were unrelated to differences between patients. Proteins related to blastocyst status with the highest fold changes were vitamin D-binding protein (0.79) and plasma serine protease inhibitor (1.4). However, both of those proteins were also detected by the Triple Quad-TOF workflow to be significant for the patient factor. Concentration values in individual samples measured by both workflows for proteins with the highest fold changes are shown in Figure 5. Most of the proteins significantly related to oocyte maturity were present at slightly higher concentrations in the group of mature oocyte outcomes (12 out of 20 proteins). Moreover, these proteins were organized in interaction clusters associated mostly with complement cascade pathway, protease inhibitor activity, hemostasis, and blood coagulation. On the other hand, most of the proteins significantly related to blastocyst status were present at slightly lower concentrations in the group of developed blastocyst outcomes (18 out of 22 proteins). These proteins also assembled in interaction clusters associated with complement cascade pathway, protein activation, defense response, and platelet degranulation.

In order to obtain a closer look into differences between studied groups, we constructed hierarchically clustered heatmaps of Pearson correlations of proteins in groups of samples considered in Table S9 using results obtained by both quantitative methods (see Figures S7–S14). Both methods generated similar representations with clearly visible differences between studied groups. Analysis of the group of immature oocyte outcomes showed a few clusters of tightly positively correlated proteins, which were negatively correlated with other clusters. On the contrary, analysis of the group of mature oocyte outcomes displayed only a few highly correlated proteins, while most of the proteins remained uncorrelated in any manner. Most of the proteins determined to be significant to oocyte maturity status (listed in Table S15) were present in correlation clusters in the analysis of immature oocyte outcomes group and absent in the analysis of mature oocyte outcomes group. Representations of blastocyst development outcomes groups are more similar; however, both positive and negative correlations are stronger in the group of undeveloped blastocysts.

## 3. Discussion

In this study, we attempted a directed development of two distinct quantitative proteomic workflows based on different high-resolution mass spectrometers: One aimed at the highest proteomic coverage (the Quad-Orbitrap workflow) and the other designed for the overall robustness of the analysis (the Triple Quad-TOF workflow). We further investigated their capabilities of hFF composition analysis and their potential to facilitate the search for oocyte quality biomarkers. Results obtained from both workflows were analyzed using TPA [15], allowing the unified comparison of the compatibility of the utilized workflows on absolute protein concentrations. The details concerning both workflows are summarized in Table 3.

The Quad-Orbitrap workflow involved the measurements on Q Exactive HF-X. Such orbitrap-based spectrometers coupled with nanoflow LC are the instruments most widely used in discovery proteomics and instruments of that type have been applied in most of the hFF proteome studies presenting high numbers of protein identifications [4,6,7,8,13]. Additionally, we combined the sensitive MS setup advantage with the most exhaustive of the tested digestion methods—MED-FASP using three consecutive digestions to increase proteomic coverage [15]. The results obtained in this study confirm the anticipated advantage of the Quad-Orbitrap workflow in both the detection and quantification of high numbers of proteins without the need of extensive fractionation (see Figure 1, Table 3). This trend is especially evident in terms of low abundance proteome components [29], which can be assumed here based on three aspects of the analysis: (i) numbers of identified proteins, (ii) numbers of quantified proteins, and (iii) numbers of statistically significant differentiators of oocyte or blastocyst status. We have shown that fractionation has a high impact on absolute concentrations of single proteins, even in the case of simple ultrafiltration (see Figure 1b) and we recommend avoiding any fractionation procedures in quantitative experiments on clinical samples.

In the Triple Quad-TOF workflow, we used another high-resolution mass spectrometer TripleTOF 5600+, which could be coupled to a more robust microflow LC, reducing the time of a single analysis and overall workload of instrument maintenance [30]. We applied SWATH-MS as a DIA method [16], which allowed us to extend the spectral library with additional fractionation while keeping the preparation of clinical samples uncomplicated. Recent developments in SWATH-MS quantification on short microflow LC gradients point to a possibility of further reduction in the analysis time [31]. Furthermore, we utilized FASP with a single trypsin digestion, which resulted in a better quantitative performance in combination with SWATH-MS than MED-FASP (see Figure 2d). Nevertheless, both FASP and MED-FASP protocols can be further shortened considerably by using reduced digestion times (1–2 h) [32], which can be cut down even more significantly with the use of the recently introduced ultrasonic-based FASP [33]. The extent of proteins identified with the Quad-Orbitrap workflow could be reached by the Triple Quad-TOF workflow only after the peptide RP-HPLC fractionation in high pH and exceeded with two-step fractionation (immunodepletion followed by HPLC). This comprehensive outcome came at a cost of additional 60 MS measurements; however, due to the principle of SWATH-MS quantification, proteins detected in the fractionation experiments could be included in the spectral library to improve quantification in unfractionated samples. The cost of additional measurements for fractionation experiments becomes less important with their application in large scale studies (on many clinical samples).

The complete separation of all stages of the proteomic analysis of clinical samples in both workflows ought to be kept in mind in the consideration of the compatibility of quantification. Due to the use of TPA, we were able to compare the compatibility of both workflows on absolute biological concentrations. In the pool unfractionated hFF sample, 309 more proteins could be quantified in the Quad-Orbitrap workflow, but only 154 more were quantified with CV less than 20% and even less—56 with CV less than 10%. This further confirms the suitability of the Quad-Orbitrap workflow for the analysis of a great number of low abundance proteome components, which is analyzed with slightly worse accuracy. The accuracy of the measurements could, however, be further improved by facilitating the concentration calculations by the implementation of the MaxLFQ algorithm [34] at the cost of lower proteomic coverage. The number of quantified proteins increases with the number of analyzed samples, but the difference is much more significant for the Triple Quad-TOF workflow. It may indicate that the Quad-Orbitrap workflow is near its limit of quantifiable proteins without fractionation, whereas the Triple Quad-TOF workflow may be capable of a moderate increase in the number of quantified proteins with larger number of samples analyzed. Proteins numbering 124 were quantified by both workflows in a pool sample. We achieved a high degree of correspondence between the workflows, which can be observed on the scatterplot in Figure 3b, and by the high Pearson correlation coefficient value of 0.86. In the set of clinical samples, the correlations between single samples analyzed by different workflows for 199 shared proteins were lower than in the case of the pool sample, yet what is noteworthy is that all samples subjected to subsequent statistical analysis (excluding the P1F2 sample) displayed Pearson correlation coefficients higher than 0.75 (see Table S12). However, in case of the analysis of single proteins in clinical samples, we found rather considerable discrepancies between both workflows as only about 20% of proteins displayed Pearson correlation higher than 0.7 in our comparison (see Table S13). The agreement of quantification was not related to the general protein concentration in hFF. On the other hand, the group quantifications of mature/immature oocyte or developed/not developed blastocyst comparisons were remarkably consistent as demonstrated by low fold change ratio differences (see Figure 4a,b). It must be noted that the overall group fold changes were rather minimal, which might have assisted the unity of both workflows in that aspect. Both workflows have shown significant similarity also in the hierarchically clustered heatmaps of Pearson correlations of proteins, which we constructed for each development status-linked group of samples (see Figures S7–S14). There, the differences between mature and immature oocyte groups and developed and not developed blastocyst groups are clearly visible due to noticeably higher numbers of positively and negatively correlated clusters in the immature oocyte/not developed blastocyst group.

Results obtained in this study point to much higher influences of inter-patient differences than the inter-follicle differences based on the status of oocyte or blastocyst on the hFF composition. Similar numbers of proteins linked to inter-patient differences have been established by both workflows; however, significantly more proteins linked to the oocyte quality features have been determined by the Quad-Orbitrap workflow. Again, it is most likely related to lower concentrations of these proteins, as for instance more than half of the proteins determined to be associated with oocyte maturity by the Quad-Orbitrap workflow have not been quantified by the Triple Quad-TOF workflow. Although only one protein per maturity comparison was appointed to be significant from both workflows (hepatocyte growth factor-like protein and carboxypeptidase B2 for oocyte and blastocyst status, respectively, see Table S14), most of the remaining significant differentiators quantified by both methods were measured with good consistency, allowing us to obtain unified lists of proteins possibly linked to oocyte quality (see Table S15). It is crucial to acknowledge the difference between the maturity comparisons carried out in this study. Identification of proteins linked to oocyte maturity is more straightforward since it is not dependent on further events, while formation of the blastocyst relies heavily on the success of the fertilization process, sperm quality, and subsequent embryo culture; factors which are separated from hFF. Thus, proteins associated with the oocyte maturity may play a more direct role in the oocyte development or their concentrations may be an explicit consequence of physiological circumstances accompanying its course. The inference of proteins related to blastocyst status, however, may be too distant or even coincidental, yet its success would be a genuine fulfillment of the oocyte quality-related investigation. List of proteins appointed by both workflows and the literature reports of their possible connection to oocyte quality are summed up in Table S15.

## 4. Materials and Methods

### 4.1. Research Approval

Conducted experiments described here are part of the “Identification of biomarkers of early embryonic development and pregnancy” project that has been approved by the Independent Bioethics Commission at the Medical University of Gdansk (decision 62/2016). Each couple undergoing IVF treatment has signed a written informed consent regarding the treatment and all included procedures. The obtained written consent also include an agreement for the publication of treatment-related data if patient anonymity is maintained.

### 4.2. Collection of Samples

A pool sample of hFF from multiple patients along with 20 samples from single follicles of four patients was obtained from the INVICTA Fertility and Reproductive Center in Sopot. Patients who took part in this study underwent the IVF procedure due to male factor infertility and received hormonal stimulation as part of the procedure. The hormonal stimulation protocol and sample retrieval procedures were outlined in detail in our previous publication [14]. All samples were free of visible blood contamination and were stored at −20 °C until analysis. Retrieved mature oocytes were fertilized by intracytoplasmic sperm injection (ICSI). Embryos were cultured as previously described [35] and evaluated according to the 2011 Istanbul consensus criteria [36].

### 4.3. Experimental Design

Specific details on the samples prepared for all the experiments described in this work are listed in Table S1, including the specification of digestion, fractionation, spiking of retention time calibration standard, mode of acquisition, instrument used, number of samples and repetitions, and the general purpose in the experimental task. Briefly, the experiments involved comparative qualitative and quantitative studies, spectral library preparation for SWATH-MS quantification, and a pilot study on clinical samples. The pilot study included five samples from single follicles per each of the four patients. Out of twenty total collected samples: five were linked to the immature oocyte and fifteen to mature oocytes out of which, after fertilization, nine developed relative to the mature blastocysts and six arrested before the compactation stage (see Table S9). Data processing for both tested workflows was performed separately for clinical samples and each pool experiment.

### 4.4. Sample Preparation

Pool samples of hFF were centrifuged at 1000× *g* for 10 min to separate cell remains from the fluid and pellets were discarded. Next, samples were either subjected to protein fractionation (Protein fractionation subsection) or left unfractionated. Protein concentrations were measured by spectrophotometer measurements of absorbance at 280 nm. The material was digested either by FASP, MED-FASP (Multi-Enzyme Digestion FASP) or in-solution digestion (see Protein digestion subsection). The resulting proteolytic peptides were fractionated by RP-HPLC in high pH (Peptide fractionation by high pH RP-HPLC subsection) or desalted in STAGE (STop And Go Extraction) Tips procedure [37] on in-house prepared tips filled with C18 solid phase (3M™ Empore™, St. Paul, MN, USA). Briefly, 10 µg of peptides was added on the tip equilibrated beforehand by 1% acetic acid in water. After washing, peptides were eluted by a buffer containing 60% acetonitrile (ACN)/1% acetic acid in water and evaporated in a SpeedVac to obtain volumes ready for MS measurements (5 µL for Q Exactive HF-X or 10 µL for Triple TOF 5600+). The iRT (indexed retention time) Kit (Biognosys, Zurich, Switzerland) was spiked into samples used for SWATH-MS spectral library preparation or SWATH-MS quantification in a 1:10 standard to sample volume ratio in order to perform the retention time calibration.

Clinical samples were prepared as described above for pooled samples; however, no protein or peptide fractionations were applied. Proteins were digested either by FASP for the Triple Quad-TOF workflow or MED-FASP for the Quad-Orbitrap workflow.

#### 4.4.1. Protein Fractionation

Samples were depleted of high abundant serum proteins (HAP) with the use of Multiple Affinity Removal Spin Cartridge (MARS Hu-14, Agilent Technologies, Santa Clara, CA, USA) according to the manufacturer’s instructions. Briefly, the material was diluted with the supplied buffer, filtered through a spin filter of 0.22 µm cutoff membrane for 2 min at 16,000× g, and applied to the cartridge according to the manual. HAP-depleted fractions and HAP-enriched fractions resulting from multiple procedures were combined individually, concentrated on 5000 kDa MW 4 mL Spin Concentrators by subsequent centrifugations at 4000× *g* 10 °C for 30 min, and finally the buffer was exchanged into 50 mM NH_4_HCO_3_ on concentrators. Both fractions were subjected to digestion.

Samples were divided into low molecular weight fraction (LMWF) and high molecular weight fraction (HMWF) by ultrafiltration on Amicon filters with 10 kDa cutoff membrane (Merck-Millipore, Burlington, MA, USA) for 15 min at 14,000 g with the addition of 20% ACN as described before [14,38]. LMWF was subsequently prepared for MS analysis in the STAGE Tips procedure. HMWF was subjected to proteolytic digestion.

#### 4.4.2. Protein Digestion

Protein material was digested by trypsin (1:50 enzyme to protein weight ratio) in a standard FASP procedure [39] on Microcon with 30 kDa cutoff membrane (Merck-Millipore, Burlington, MA, USA). The MED-FASP procedure involved three consecutive digestions by LysC (1:50), trypsin (1:100), and chymotrypsin (1:100); and the modified MED-FASP [39] consisted of two digestions: trypsin (1:50) and chymotrypsin (1:100) (all enzymes from Promega Corporation, Madison, WI, USA). First, hFF was lysed with the use of buffer containing 1% sodium dodecyl sulfate (SDS), 50 mM dithiothreitol (DTT) in 100 mM Tris-HCl pH 8 for 10 min in 95 °C (all reagents from Sigma-Aldrich, St. Louis, MO, USA). The amount of 100 µg of protein was administered to each filter. Briefly, the filters were washed with the buffer containing 8 M urea in 100 mM Tris-HCl pH 8.5 multiple times by centrifugation at 10,000× *g* in 20 min. Proteins were alkylated with the use of 55 mM iodoacetamide (IAA, Sigma-Aldrich, St. Louis, MO, USA) at room temperature in the dark for 20 min. Finally, traces of IAA and urea were washed with 100 mM Tris-HCl pH 8.5 and enzyme was added to the filters for the overnight digestion at 37 °C. The resulting peptides were eluted with 100 mM Tris-HCl pH 8.5. In the case of MED-FASP, filters were placed in new tubes and digestion and elution steps were repeated with other enzymes. Digestion with chymotrypsin was performed for 3 h in the buffer containing 10 mM CaCl_2_ in 100 mM Tris-HCl pH 7.8. Eluted peptides were desalted in the STAGE Tips procedure [37].

In-solution protein digestion was executed according to previously used protocol [14,40] suggested by Gundry et al. [41]. Briefly, proteins diluted in 50 mM NH_4_HCO_3_ solution were subjected to reduction by 10 mM DTT for 30 min at 56 °C and subsequent alkylation by 20 mM IAA for 30 min at room temperature. Trypsin in 1:50 enzyme to protein weight ratio was added to samples for overnight incubation at 37 °C. Digestion was stopped by 50% ACN/5% trifluoroacetic acid solution in water and samples were desalted in the STAGE Tips procedure [37].

#### 4.4.3. Peptide Fractionation by High pH RP-HPLC

Non-desalted samples of peptide material were fractionated by RP-HPLC separations in high pH in a similar manner as described before [40] on the analytical Prominence HPLC System with the UV-VIS detector (Shimadzu, Kyoto, Japan). Applied buffer system consisted of Buffer A: 0.1% NH_4_OH in water, pH 10; B: 0.1% NH_4_OH in ACN, pH 10. The amount of 1 mg of peptides was separated on the Zorbax Extend-C18 column (4.6 × 150 mm, 5 μm particle size, Agilent Technologies, Santa Clara, CA, USA) into 60 2 mL fractions in 120 min gradient (0–40% Buffer B in 100 min followed by 40–100% Buffer B in 20 min). All collected fractions were evaporated to dryness in a SpeedVac, dissolved in 60% ACN/1% acetic acid in water and again evaporated to a volume of 10 µL, with the exception of 2 first fractions from each separation which were desalted in the STAGE Tips procedure [37] due to salt accumulation in those fractions.

### 4.5. LC-MS/MS Measurements and Quantitative Data Processing

#### 4.5.1. Triple Quad-TOF Workflow

The LC-MS/MS measurements for the Triple Quad-TOF workflow were acquired on the TripleTOF 5600+ hybrid mass spectrometer with DuoSpray Ion Source (AB SCIEX, Framingham, MA, USA) coupled with the Eksigent microLC (Ekspert MicroLC 200 Plus System, Eksigent, Redwood City, CA, USA) in a similar manner as described before [14,40]. Samples were loaded onto the LC column using the CTC Pal Autosampler (CTC Analytics AG, Zwinger, Switzerland), with a 5 µL injection. The Buffers A and B constituted of 0.1% (v/v) formic acid in water and ACN, respectively. LC separations were carried out on the ChromXP C18CL column (3 μm, 120 Å, 150 × 0.3 mm; Eksigent, Redwood City, CA, USA) using a gradient of 8–40% Buffer B in 30 min with a flowrate of 5 µL/min. All measurements were conducted in a positive ion mode. The system was controlled by the Analyst TF 1.7.1 software (AB SCIEX, Framingham, MA, USA). The data-dependent acquisition (DDA) analyses consisted of a 250 ms TOF survey scan in the m/z range of 400–1000 Da followed by a 100 ms Product Ion scan in the m/z range of 100–1500 Da, which resulted in a 2.3 s cycle time. Top 20 candidate ions with charge state of 2 to 5 were selected for collision-induced dissociation (CID) fragmentation with a rolling collision energy. Former target ions were excluded after 2 occurrences for 5 s. SWATH-MS [16] analyses were performed in a looped product ion mode. A set of 25 variable-width windows were constructed by equalized ion frequency distribution with the use of SWATHTuner [42] to cover the m/z range of 400–1000 Da. The collision energy for each window was calculated for +2 to +5 charged ions centered upon the window with a spread of 5. The SWATH-MS1 survey scan was acquired in high sensitivity mode in the range of 400–1000 Da in the beginning of each cycle with the accumulation time of 50 ms and it was followed by 40 ms accumulation time high sensitivity product ion scans, which resulted in the total cycle time of 1.1 s. The database search for the construction of spectral library (see Experimental Design section) was performed in ProteinPilot 4.5 Software (AB SCIEX, Framingham, MA, USA) using the Paragon algorithm against the SwissProt *Homo sapiens* database (ver. 26.07.2019; 20,428 entries) merged with iRT standard sequence and the following parameters: TripleTOF 5600 instrument; alkylation of cysteines by iodoacetamide; trypsin enzyme digestion, ID focus on biological modifications; search effort “thorough ID”; and detected protein threshold [Conf] > 10%. The resulting group file was loaded into MS/MS All with SWATH Acquisition MicroApp 2.01 in PeakView 2.2 (AB SCIEX, Framingham, MA, USA) to automatically create a spectral library with the set parameters: modified peptides allowed and shared peptides excluded. The library was processed with SWATH-MS measurements of either the pool samples or the clinical samples (see Experimental Design section). The retention time calibration was performed manually with the use of iRT kit peptides. The maximum number of peptides per protein was 6 and extracted ion chromatogram (XIC) parameters were set to 10 min extraction window width and 75 ppm XIC width. Absolute concentration values were derived from the SWATH-MS intensities using the Total Protein Approach [15].

#### 4.5.2. Quad-Orbitrap Workflow

The LC-MS/MS measurements for the Quad-Orbitrap workflow were acquired on the QExactive HF-X mass spectrometer (ThermoFisher Scientific, Palo Alto, CA, USA) coupled with nanoLC in a similar manner as described before [43]. LC separations were conducted on a 50 cm column packed with C18 material with 75 µm inner diameter in an ACN gradient of 5–30% in 95 min at the flowrate of 0.3 µL/min. The mass spectrometer operated in DDA mode with survey scans acquired at a resolution of 60,000. Top 15 ions with charge ≥ +2 were selected from the survey scan in the range of 300–1650 m/z with an isolation window of 1.4 m/z and fragmented by high-energy collision dissociation (HCD) with normalized collision energies of 25. The dynamic exclusion time was 30 s. The maximum ion injection times for the survey scan and the MS/MS scans were 20 and 28 ms, respectively. The ion target value for MS1 and MS2 scan modes was set to 3 × 10^6^ and 10^5^, respectively. The raw spectra of either all pool samples or all clinical samples (see Experimental Design section) were processed in the MaxQuant software [44], version 1.6.2.6a against the SwissProt *Homo sapiens* database (see Triple Quad-TOF workflow subsection) with the following parameters: carbamidomethylation as a fixed modification, trypsin enzyme digestion, and 0.01 false discovery rate (FDR). Quantification of protein concentrations was performed using the Total Protein Approach [15] for proteins with a minimum of 2 unique peptides and 70% valid values across the measurements.

### 4.6. Data Analysis

Database search of DDA runs was conducted with ProteinPilot and MaxQuant software for Triple Quad-TOF and Quad-Orbitrap workflows, respectively. The specific search settings in all database search engines were dependent on the experiment (see Table S1) in each software with respect to the following aspects: proteolytic digestion (LysC, trypsin, trypsin + chymotrypsin, or no digestion) and fixed modifications (carbamidomethylation of cysteines or none). All results were filtered for 0.01 FDR at the protein level. Functional analysis of proteins and interaction network construction was performed using STRING database [18], version 11. Network visualization was conducted in Cytoscape 3.8.2 [45]. Venn plots were constructed using the online Venny 2.1 tool [46]. Bar plots were created in Microsoft Excel. Additional calculations and plot visualizations, including Pearson correlation calculation and hierarchically clustered heatmaps, were performed using Python 3.8 including libraries: numpy 1.18.5, pandas 1.1.1, matplotlib 3.3.1, and seaborn 0.11.0. The mass spectrometry proteomics data have been deposited to the ProteomeXchange Consortium [47] via the PRIDE [48] partner repository with two dataset identifiers PXD024223 and PXD024347 for projects divided into data acquired with the Triple Quad-TOF workflow and Quad-Orbitrap workflow, respectively.

### 4.7. Statistical Analysis

Statistical analysis was performed using Perseus 1.6.14.0 [49]. Replicates were averaged by the median value. Data were log2-transformed, any missing values were imputed from normal distribution, inspected using scatterplots, and normalized by z-score. Two separate two-way ANOVA analyses were performed with one factor grouping samples from a single patient and another factor grouping samples associated with the mature/immature oocyte or developed/not developed blastocyst. Post hoc Tukey’s HSD tests were performed for each factor in both analyses on 0.05 and 0.01 FDR level.

## 5. Conclusions

We have developed and presented here results of tests of two completely separate quantitative proteomic workflows involving the use of different MS instruments and analyzed their compatibility in absolute quantification of hFF proteins with the use of TPA. We observed remarkably high correlation between analyses of single samples (see Figure 3b, Table S12) and moderate correlation between analyses of single proteins (see Figure S4, Table S13) obtained by applying the Quad-Orbitrap and Triple Quad-TOF workflows. Moreover, the final biological information was widely consistent. We have obtained combined lists of proteins possibly associated with oocyte quality: 20 proteins linked to oocyte maturity and 22 proteins linked to blastocyst development status (see Table S15). Demonstrated here are proteins linked to oocyte or blastocyst status that may pose as primary targets for the candidates of the oocyte quality which should be verified on a larger set of samples.

Both tested workflows are interchangeable in general terms of the obtained results; however, each workflow has its own advantages and limitations. The Quad-Orbitrap workflow (see Table 3) was indisputably best suited for in-depth proteomic analysis of protein targets present in a wide range of physiological concentrations, especially low abundant proteome; however, high sensitivity comes at a price of longer LC-MS/MS measurement time and the cost of digestion protocol. The use of the less effective Triple Quad-TOF workflow in terms of numbers of identified and quantified proteins (see Table 3) comes with a significant advantage of more than three times shorter LC-MS/MS analysis time as well as shorter and less expensive sample preparation protocol (one enzyme used in digestion instead of three) in comparison to the Quad-Orbitrap workflow. It was already mentioned that both workflows have a potential for further analysis time reduction as well as the increase in their detection and quantification capacity with the number of analyzed samples, which is more evident in the case of the Triple Quad-TOF workflow (see Discussion). Since the results obtained by both workflows are well correlated, a choice of the workflow for a given task should be steered by its limitations and advantages as well as the access to a given type of equipment.

## Figures and Tables

**Table 1:** Sample preparation scheme including protein fractionation, method of digestion, peptide fractionation, and instrument used in each described experiment. Fraction analyzed designates the material, which was further prepared or analyzed as described, resulting from a given fractionation technique. More details on sample preparation of single samples are included in Table S1.

Workflow Abbreviation	Protein Fractionation	Fraction Analyzed	Digestion	Peptide Fractionation	Fraction Analyzed	Instrument
**3TOF HFF T**	None	-	FASP with trypsin	None	-	Triple TOF 5600+
**3TOF HMW T**	Ultrafiltration	Retentate (>10 kDa)	FASP with trypsin	None	-	Triple TOF 5600+
**3TOF MF T**	Immunodepletion	Low abundant proteins (MARS-14)	FASP with trypsin	None	-	Triple TOF 5600+
**3TOF MR T**	Immunodepletion	High abundant proteins (MARS-14)	FASP with trypsin	None	-	Triple TOF 5600+
**3TOF HPLC1**	None	-	FASP with trypsin	High pH RP-HPLC	60 separate fractions	Triple TOF 5600+
**3TOF HPLC2**	Immunodepletion	Low abundant proteins (MARS-14)	FASP with trypsin	High pH RP-HPLC	60 separate fractions	Triple TOF 5600+
**3TOF HFF LTC**	None		MED-FASP (LysC, trypsin, and chymotrypsin)	None	-	Triple TOF 5600+
**3TOF HFF TC**	None	-	MED-FASP (trypsin and chymotrypsin)	None	-	Triple TOF 5600+
**3TOF HFF IS**	None	-	In solution with trypsin	None	-	Triple TOF 5600+
**3TOF LMW**	Ultrafiltration	Filtrate (<10 kDa)	None	None	-	Triple TOF 5600+
**QE HFF LTC**	None	-	MED-FASP (LysC, trypsin, and chymotrypsin)	None	-	Q Exactive HF-X
**QE HMW LTC**	Ultrafiltration	Retentate (>10 kDa)	MED-FASP (LysC, trypsin, and chymotrypsin)	None	-	Q Exactive HF-X
**QE MF LTC**	Immunodepletion	Low abundant proteins (MARS-14)	MED-FASP (LysC, trypsin, and chymotrypsin)	None	-	Q Exactive HF-X
**QE MR LTC**	Immunodepletion	High abundant proteins (MARS-14)	MED-FASP (LysC, trypsin, and chymotrypsin)	None	-	Q Exactive HF-X
**QE HFF IS**	None	-	In solution with trypsin	None	-	Q Exactive HF-X
**QE LMW**	Ultrafiltration	Filtrate (<10 kDa)	None	None	-	Q Exactive HF-X

**Table 2:** Numbers of proteins identified in this study by Quad-Orbitrap (QE) or Triple Quad-TOF (3TOF) workflows, which were also reported in proteomic studies of hFF or proximate biological materials (plasma, oocyte, and granulosa cell).

	All Identified Proteins		All Proteins Identified in HMW Fraction		Proteins Identified only in HMW Fraction		All Proteins Identified in LMW Fraction		Proteins Identified only in LMW Fraction	
Resource	QE (942)	3TOF (1182)	QE (873)	3TOF (1177)	QE (785)	3TOF (1168)	QE (157)	3TOF (14)	QE (69)	3TOF (5)
Plasma Proteome Database [21]	773	975	723	975	644	966	129	9	50	0
Human oocyte [1] (oocyte specific)	226 (22)	301 (18)	211 (22)	301 (18)	183 (18)	294 (17)	43 (4)	7 (1)	15	0
Human granulosa cell [22]	436	599	390	599	346	591	90	8	46	0
HFF (Zamah et al., 2015) [6]	545	610	542	610	470	602	75	8	3	0
HFF (Bianchi et al., 2016) [5]	357	368	352	368	284	360	73	8	5	0
HFF (Oh et al., 2017) [7]	521	534	518	534	445	525	76	9	3	0
HFF (Poulsen et al., 2019) [4]	336	330	333	330	269	321	67	9	3	0
HFF (Zhang et al., 2019) [9]	567	647	540	647	463	640	104	7	27	0
HFF from hSAF (Pla et al., 2020) [10]	829	987	794	987	708	978	121	9	35	0
Unique	28	40	20	35	20	35	8	5	8	5

Identifications were divided into HMW and LMW fractions and identifications specific to each fraction were additionally separated. Numbers of total identifications in each instance are given in brackets. The proteomic study of human oocyte contained an inferred set of proteins specific to the oocyte; the comparison to this set is given in brackets. Numbers termed as unique denote proteins identified in this study and not reported in any listed resource.

**Table 3:** General description of the components and main outcomes of the two applied protein quantification workflows.

	Method 1 (Quad-Orbitrap Workflow)	Method 2 (Triple Quad-TOF Workflow)
Instrument	Q Exactive HF-X	TripleTOF 5600+
LC (flowrate)	nanoflow (0.3 µL/min)	microflow (5 µL/min)
Digestion method (enzymes)	MED-FASP (Lys-C, Trypsin, Chymotrypsin)	FASP (Trypsin)
Quantification method	DDA-TPA	DIA-TPA (SWATH-MS based)
Single LC-MS/MS analysis time (full in triplicate)	95 min (285 min)	30 min (90 min)
Number of proteins identified in the unfractionated sample using the described digestion method	565/380 (MED-FASP/IS)	129/154/93 (FASP/MED-FASP/IS)
Number of proteins identified in the LMW fraction	157	14
Number of proteins quantified in the pool sample	438	129
Number of proteins quantified in the pool sample with CV < 10%/< 20%	126/252	70/98
Number of proteins quantified in the clinical samples	455	215
Number of proteins linked to the patient factor (inter-patient differences) at 1%/5% FDR	68/101	64/96
Number of proteins linked to the oocyte status (inter-follicle differences) at 1%/5% FDR	11/49	1/10
Number of proteins linked to the blastocyst status (inter-follicle differences) at 1%/5% FDR	13/45	2/7

## Data Availability

The mass spectrometry proteomics data have been deposited to the ProteomeXchange Consortium [47] via the PRIDE [48] partner repository with two dataset identifiers PXD024223 and PXD024347.

## Supplementary Materials

The following are available online at https://www.mdpi.com/article/10.3390/ijms22147415/s1, Supporting Material 1, Figure S1: Interaction networks of proteins identified in LMW fraction generated in STRING, Figures S2 and S3: Multiscatter plots displaying correlations of all clinical samples in quantitative workflows, Figure S4: Pearson correlation of concentration changes measured by both quantitative methods for each protein in the full set of samples and in the set excluding the P1F2 sample, Figure S5: Proteins determined as statistically significant by two-factor ANOVA for the factor relating to inter-patient differences in each comparison (patient-oocyte status and patient-blastocyst status) for both quantitative methods, Figure S6: Interaction networks of all proteins determined as statistically significant by two-factor ANOVA for the factor relating to oocyte maturity or blastocyst status for the Quad-Orbitrap or Triple Quad-TOF workflow at 5% FDR (PDF); Supporting Material 2, Table S1: Detailed description of the purpose and analysis workflow of all samples described in this study, Table S2: List of all proteins identified by the Quad-Orbitrap workflow in pool experiments described in detail in Table S1, Table S3: The results of quantitative analysis of unfractionated pool samples by the Quad-Orbitrap workflow along with TPA absolute concentration and coefficient of variation calculation, Tables S4 and S5: Functional enrichment analysis of proteins identified in HMWF and LMWF, Table S6: List of all proteins identified by the Triple Quad-TOF workflow in pool experiments described in detail in Table S1, Table S7: The results of quantitative analysis of unfractionated pool samples by the Triple Quad-TOF workflow along with TPA absolute concentration and coefficient of variation calculation, Table S8: List of all proteins identified in the study with the literature comparison to proteomic studies of hFF and related biological materials, Table S9: Assignment of clinical samples to analyzed groups, Tables S10 and S11: The results of quantitative analysis of clinical samples by both workflows, Table S12: Pearson correlation calculated for each sample comparison in both workflows, Table S13: Pearson correlation for each protein in both workflows for all clinical samples and excluding the P1F2 sample, Table S14: The results of all conducted two-way ANOVA tests for both workflows, Table S15: List of proteins determined to be statistically significant in relation to oocyte or blastocyst status by any applied quantification method, with less than 20% difference in median fold change ratio established by both methods (XLSX); Supporting Material 3, Figures S7–S14: Hierarchically clustered heatmaps of Pearson correlations of proteins in groups of samples considered in Table S10 using results obtained by both quantitative workflows (PDF).
