# Supplementary material for: Compatibility of Distinct Label-Free Proteomic Workflows in Absolute Quantification of Proteins Linked to the Oocyte Quality in Human Follicular Fluid
Source: Int J Mol Sci. 2021 Jul 10;22(14):7415. doi: 10.3390/ijms22147415 (PMC8304916; doi:10.3390/ijms22147415)
Supplement: Supplementary file 1 [file SupportingMaterial3.pdf › SupportingMaterial3.pdf]

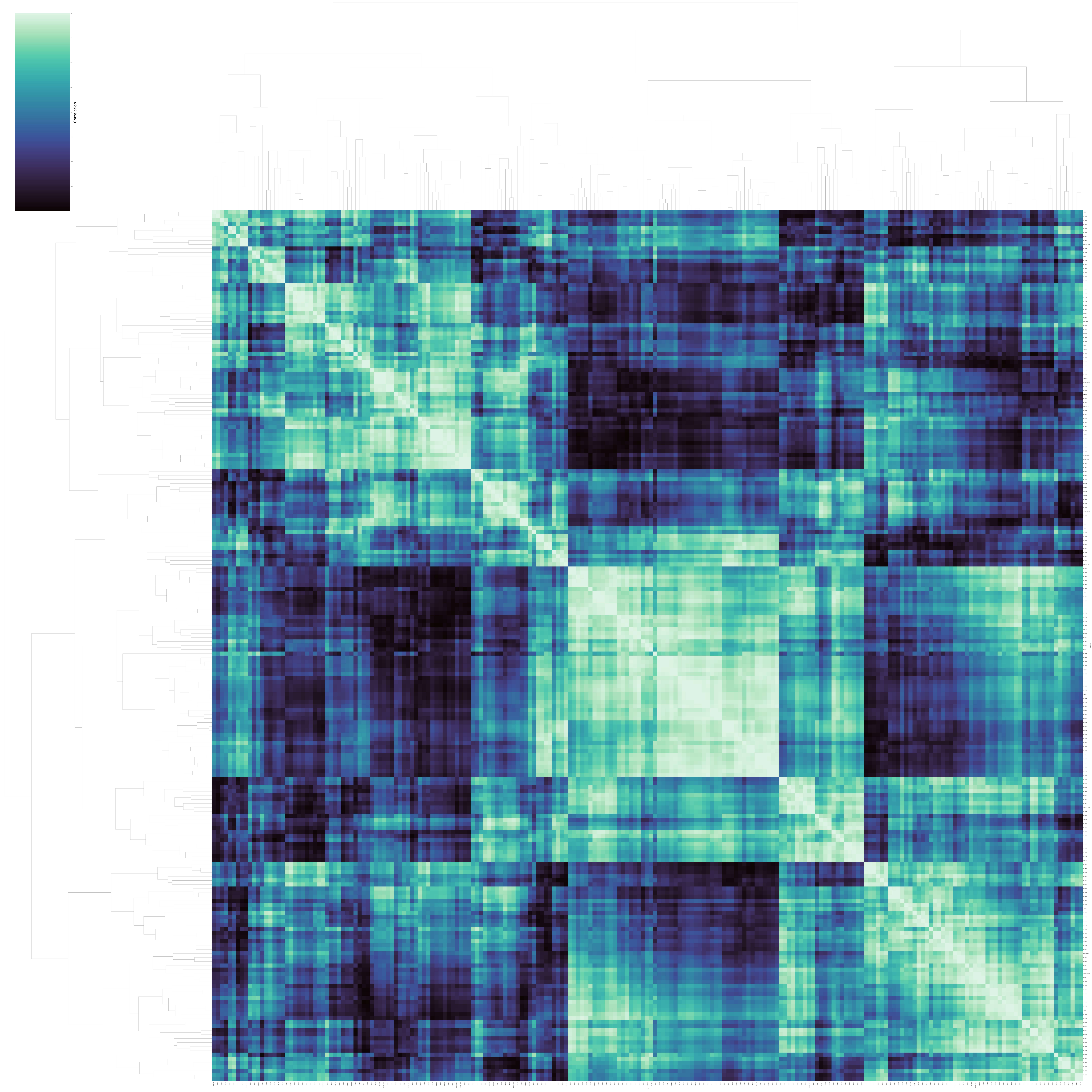

Figure S7. Hierarchically clustered heatmap of Pearson correlation coefficients of proteins in the immature oocyte group of samples using results obtained by the Triple Quad-TOF workflow.

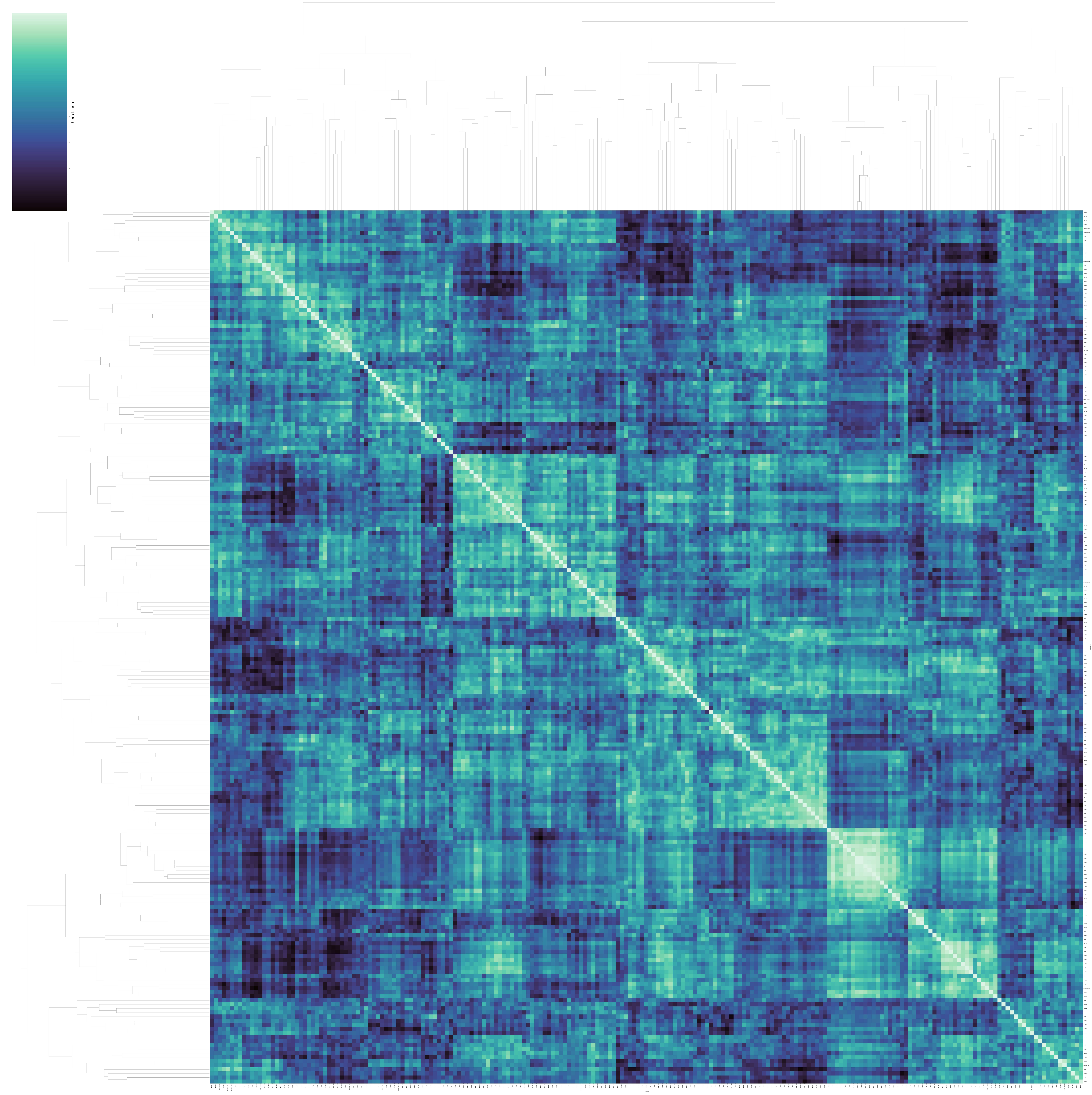

Figure S8. Hierarchically clustered heatmap of Pearson correlation coefficients of proteins in the mature oocyte group of samples using results obtained by the Triple Quad-TOF workflow.

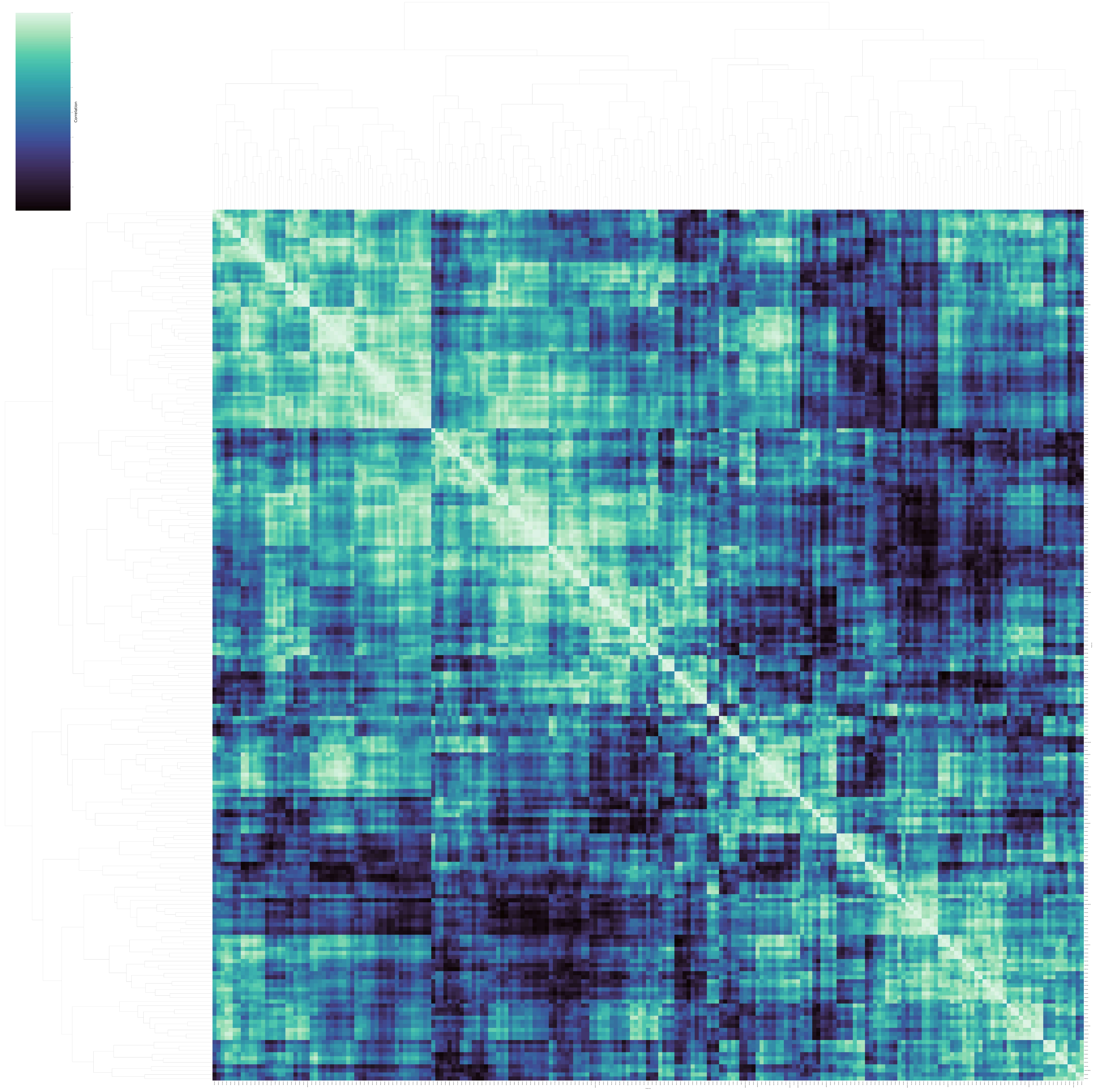

Figure S9. Hierarchically clustered heatmap of Pearson correlation coefficients of proteins in the not developed blastocyst group of samples using results obtained by the Triple Quad-TOF workflow.

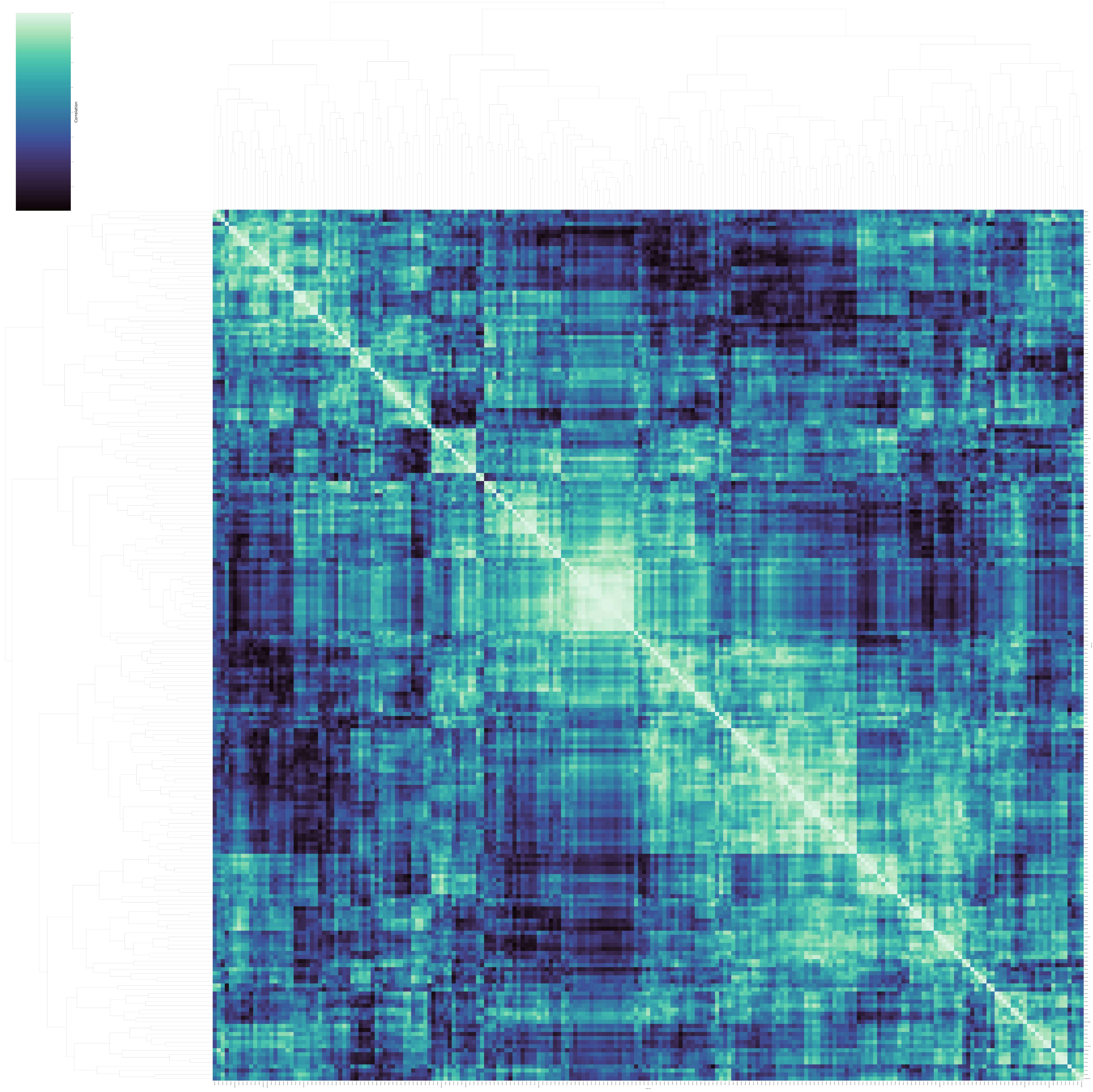

Figure S10. Hierarchically clustered heatmap of Pearson correlation coefficients of proteins in the developed blastocyst group of samples using results obtained by the Triple Quad-TOF workflow.

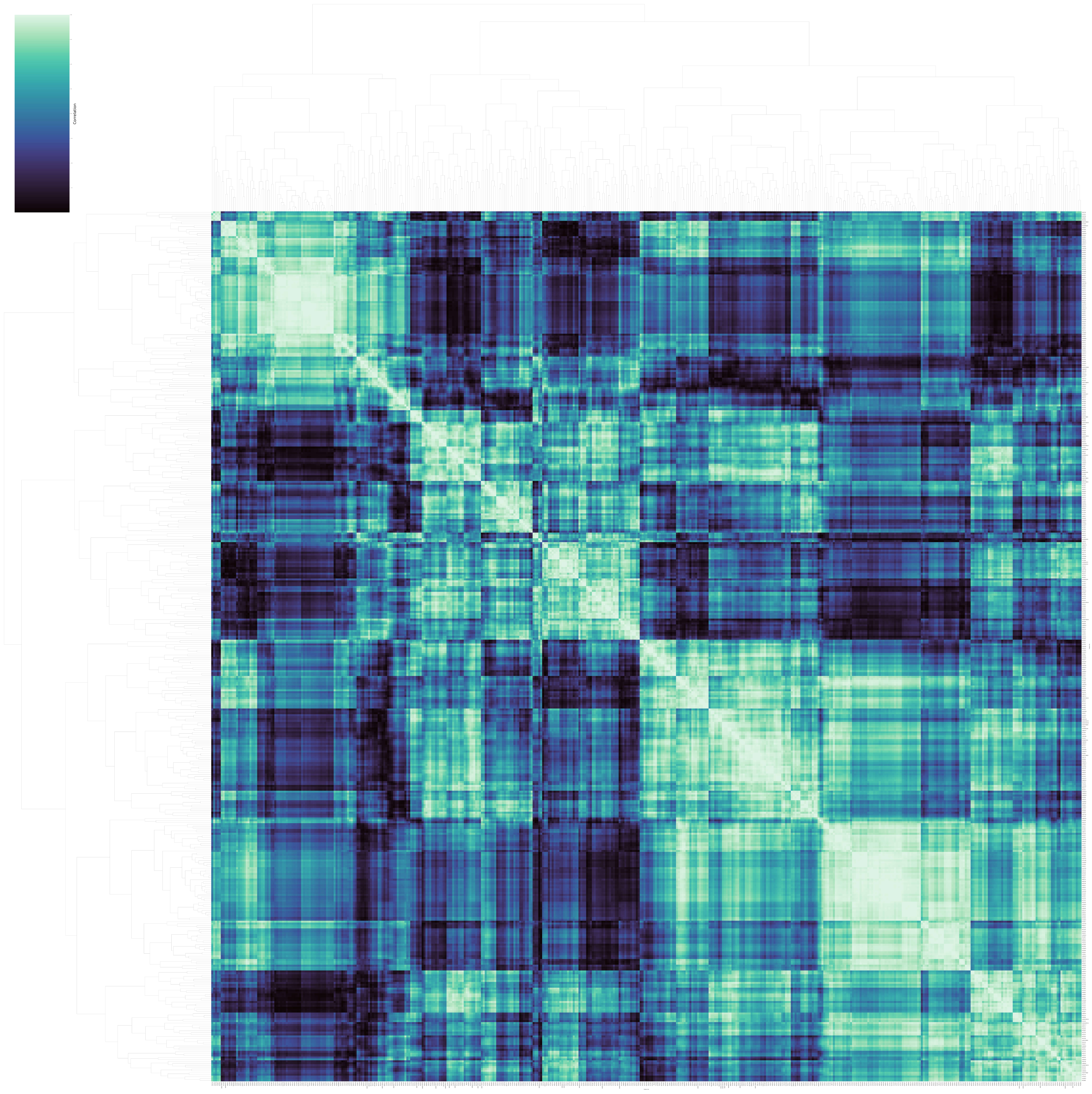

Figure S11. Hierarchically clustered heatmap of Pearson correlation coefficients of proteins in the immature oocyte group of samples using results obtained by the Quad-Orbitrap workflow.

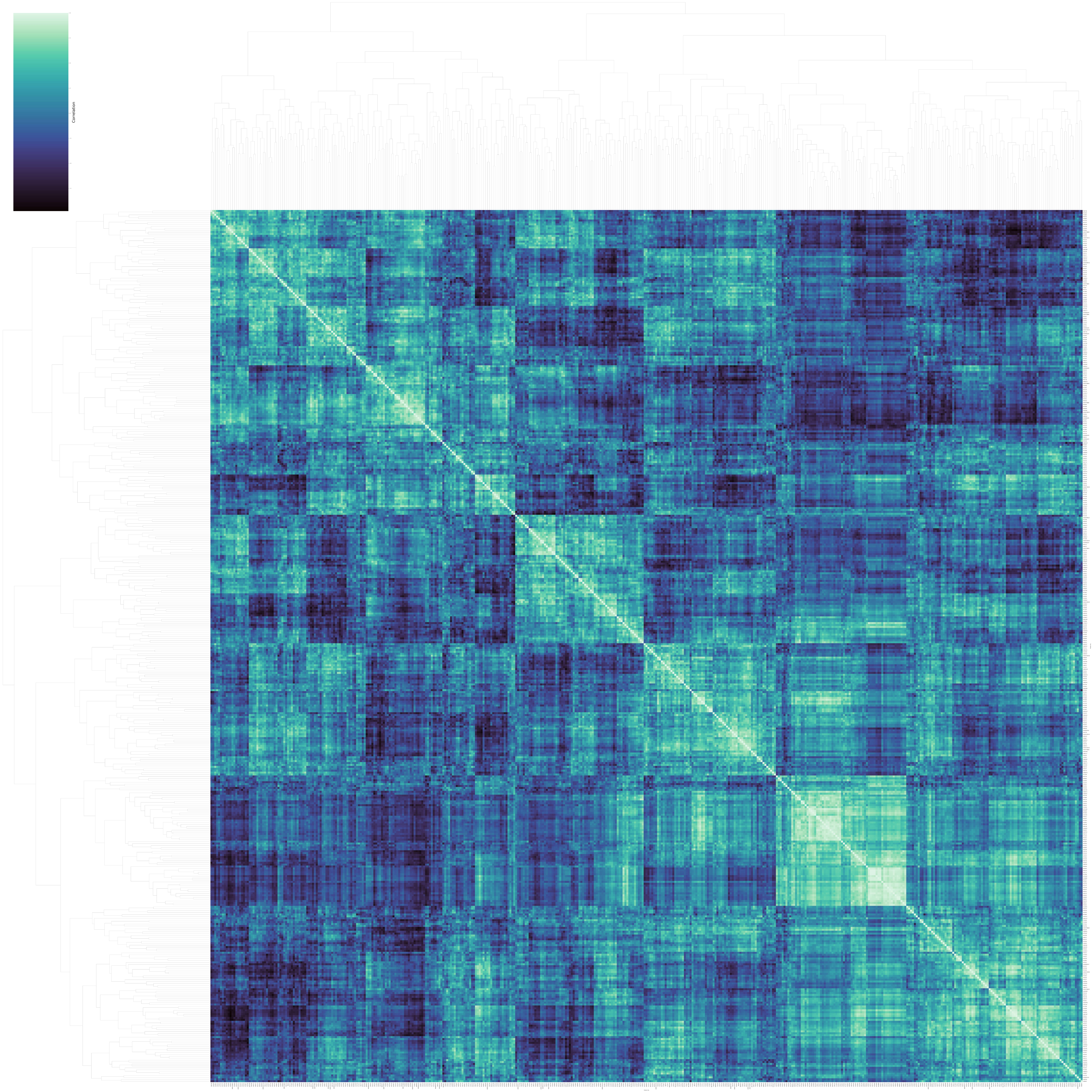

Figure S12. Hierarchically clustered heatmap of Pearson correlation coefficients of proteins in the mature oocyte group of samples using results obtained by the Quad-Orbitrap workflow.

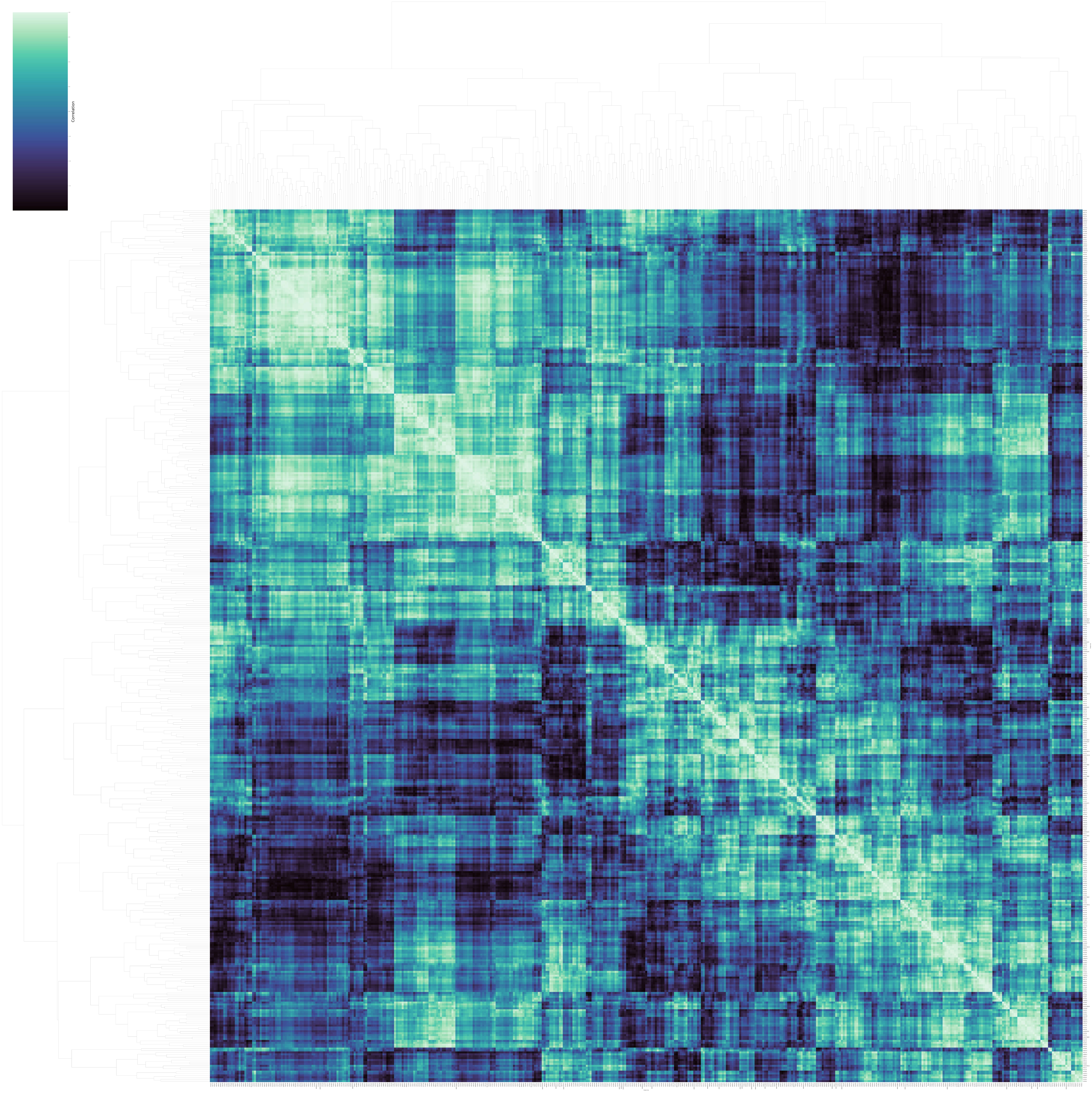

Figure S13. Hierarchically clustered heatmap of Pearson correlation coefficients of proteins in the not developed blastocyst group of samples using results obtained by the Quad-Orbitrap workflow.

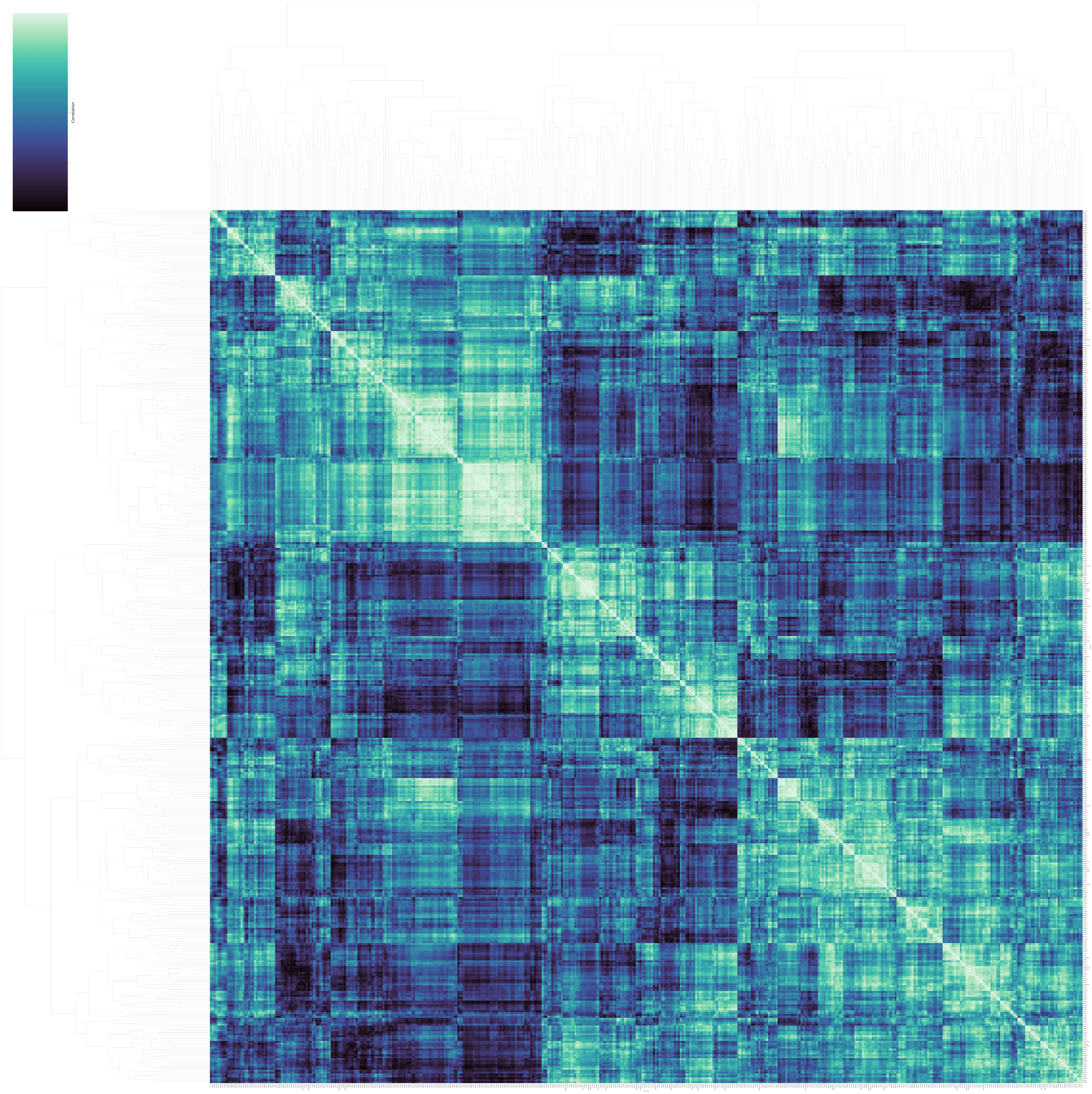

Figure S14. Hierarchically clustered heatmap of Pearson correlation coefficients of proteins in the developed blastocyst group of samples using results obtained by the Quad-Orbitrap workflow.
