# Supplementary material for: Compatibility of Distinct Label-Free Proteomic Workflows in Absolute Quantification of Proteins Linked to the Oocyte Quality in Human Follicular Fluid
Source: Int J Mol Sci. 2021 Jul 10;22(14):7415. doi: 10.3390/ijms22147415 (PMC8304916; doi:10.3390/ijms22147415)
Supplement: Supplementary file 1 [file ijms-22-07415-s001.zip › SupportingMaterial1.pdf]

Compatibility of distinct label-free proteomic workflows in determination of proteins linked to the oocyte quality in human follicular fluid

Aleksandra E. Lewandowska[*,1], *Anna Fel*[1], *Marcel Thiel*[1], *Paulina Czaplewska*[1], *Krzysztof Łukaszuk*[2,3], *Jacek R. Wiśniewski*[4], *Stanisław Ołdziej*[*,1]

1 Intercollegiate Faculty of Biotechnology UG&MUG, University of Gdańsk, Abrahama 58, 80-307 Gdańsk, Gdańsk, Poland.

2 INVICTA Fertility and Reproductive Center, Polna 64, 81-740 Sopot, Sopot, Poland.

3 Department of Obstetrics and Gynecological Nursing, Faculty of Health Sciences, Medical University of Gdańsk, Dębinki 7, 80-211 Gdańsk, Gdańsk, Poland.

4 Department of Proteomics and Signal Transduction, Max-Planck-Institute of Biochemistry, Am Klopferspitz 18, 82152 Martinsried, Germany.

* Corresponding author

Supporting Information

TABLE OF CONTENTS:

Supporting Material 1 (PDF file):

Figure S1. Interaction networks of proteins identified in LMW fraction generated in STRING.

Figure S2, 3. Multiscatter plots displaying correlations of all clinical samples in quantitative workflows.

Figure S4. Pearson correlation of concentration changes measured by both quantitative methods for each protein in the full set of samples and in the set excluding the P1F2 sample.

Figure S5. Proteins determined as statistically significant by two-factor ANOVA for the factor relating to inter-patient differences in each comparison (patient-oocyte status and patient-blastocyst status) for both quantitative methods.

Figure S6. Interaction networks of all proteins determined as statistically significant by two-factor ANOVA for the factor relating to oocyte maturity or blastocyst status for the Quad-Orbitrap or Triple Quad-TOF workflow at 5% FDR.

Supporting Material 2 (XLSX file):

Table S1. Detailed description of the purpose and analysis workflow of all samples described in this study.

Table S2. List of all proteins identified by the Quad-Orbitrap workflow in pool experiments described in detail in Table S1.

Table S3. The results of quantitative analysis of unfractionated pool samples by the Quad-Orbitrap workflow along with TPA absolute concentration and coefficient of variation calculation.

Table S4,5. Functional enrichment analysis of proteins identified in HMWF and LMWF.

Table S6. List of all proteins identified by the Triple Quad-TOF workflow in pool experiments described in detail in Table S1.

Table S7. The results of quantitative analysis of unfractionated pool samples by the Triple Quad-TOF workflow along with TPA absolute concentration and coefficient of variation calculation.

Table S8. List of all proteins identified in the study with the literature comparison to proteomic studies of hFF and related biological materials.

Table S9. Assignment of clinical samples to analyzed groups.

Table S10,11. The results of quantitative analysis of clinical samples by both workflows.

Table S12. Pearson correlation calculated for each sample comparison in both workflows.

Table S13. Pearson correlation of both workflows for all clinical samples and excluding the P1F2 sample.

Table S14. The results of all conducted two-way ANOVA tests for both workflows.

Table S15. List of proteins determined to be statistically significant in relation to oocyte or blastocyst status by any applied quantification method, with less than 20% difference in median fold changes established by both methods.

Supporting Material 3 (PDF file):

Figures S7-14. Hierarchically clustered heatmaps of Pearson correlations of proteins in groups of samples considered in Table S9 using results obtained by both quantitative workflows.

(a)

(b)

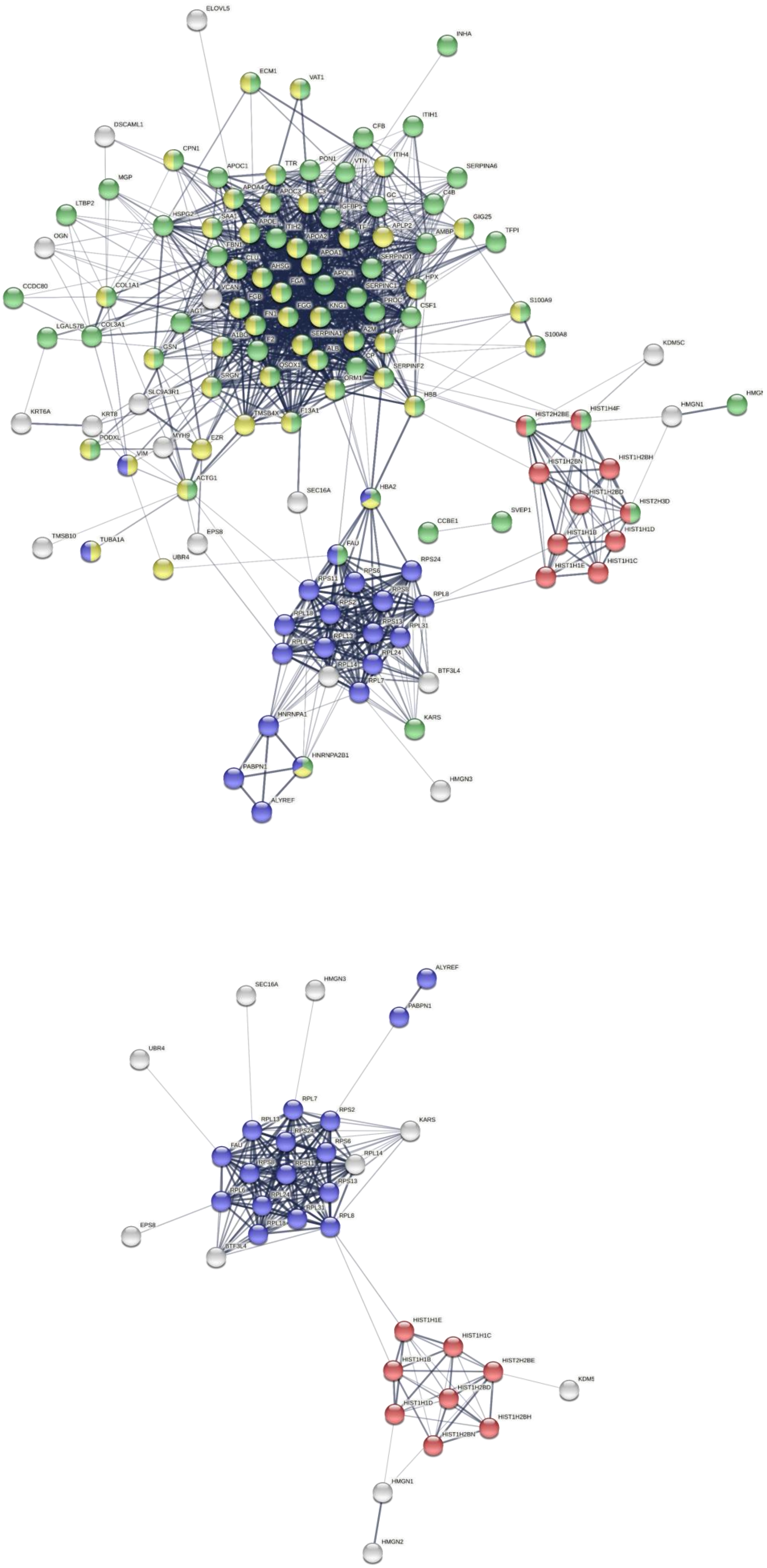

Figure S1. Interaction networks of proteins identified by the Quad-Orbitrap workflow in the LMW fraction generated in STRING: all identified proteins **(a)**, proteins identified exclusively in LMWF **(b)**. Nodes are colored according to their assignment to cellular component from Gene Ontology database: nucleosome (red), ribonucleoprotein complex (blue), extracellular region (green), and vesicle (yellow). Non-interacting proteins not shown.

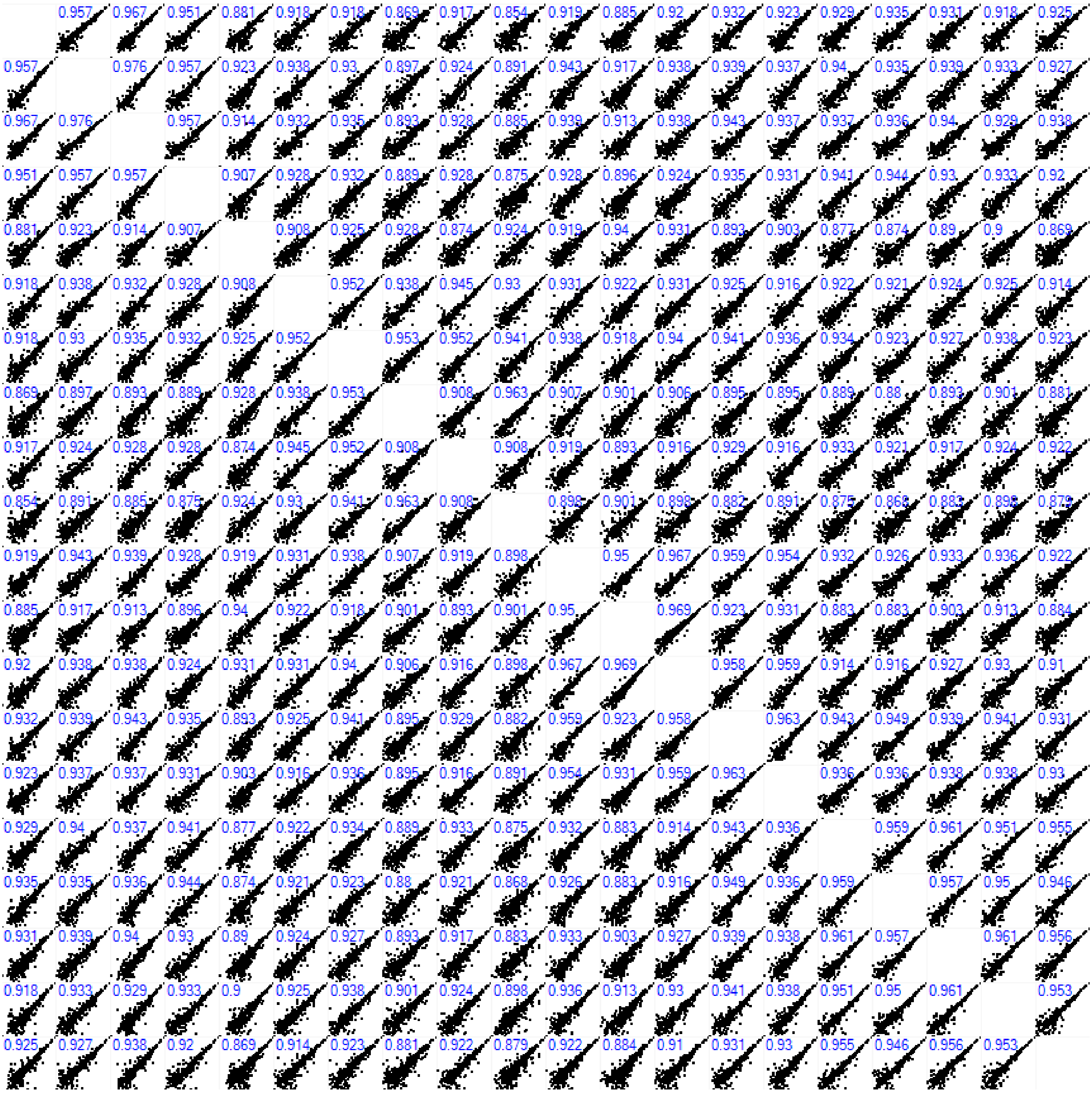

Figure S2. Multiscatter plots displaying correlations of all clinical samples analyzed by the Quad-Orbitrap workflow. Quantitative values were log2-transformed and missing values were imputed from normal distribution. Pearson correlations are shown for each scatter plot.

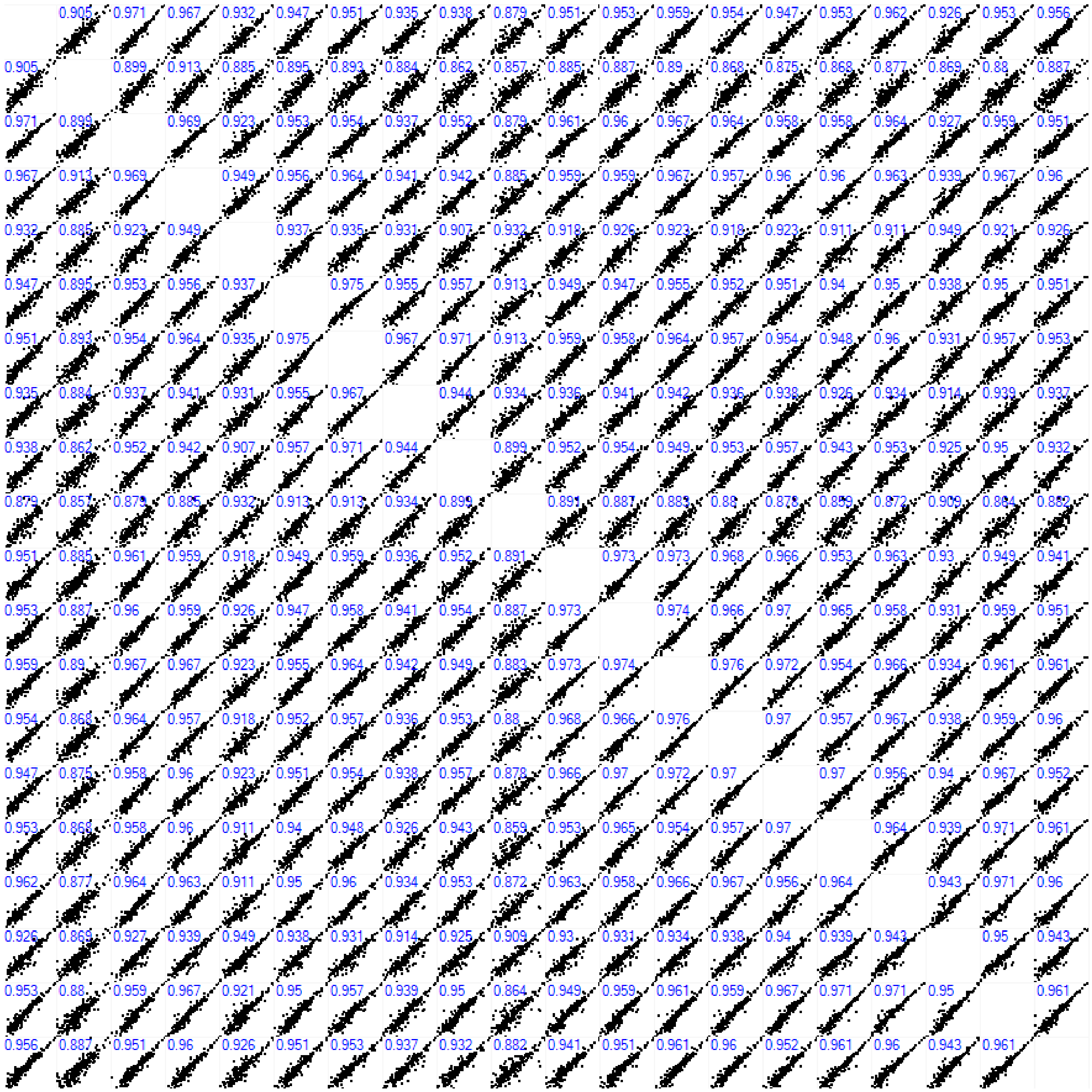

Figure S3. Multiscatter plots displaying correlations of all clinical samples analyzed by the Triple Quad-TOF workflow. Quantitative values were log2-transformed and missing values were imputed from normal distribution. Pearson correlations are shown for each scatter plot.

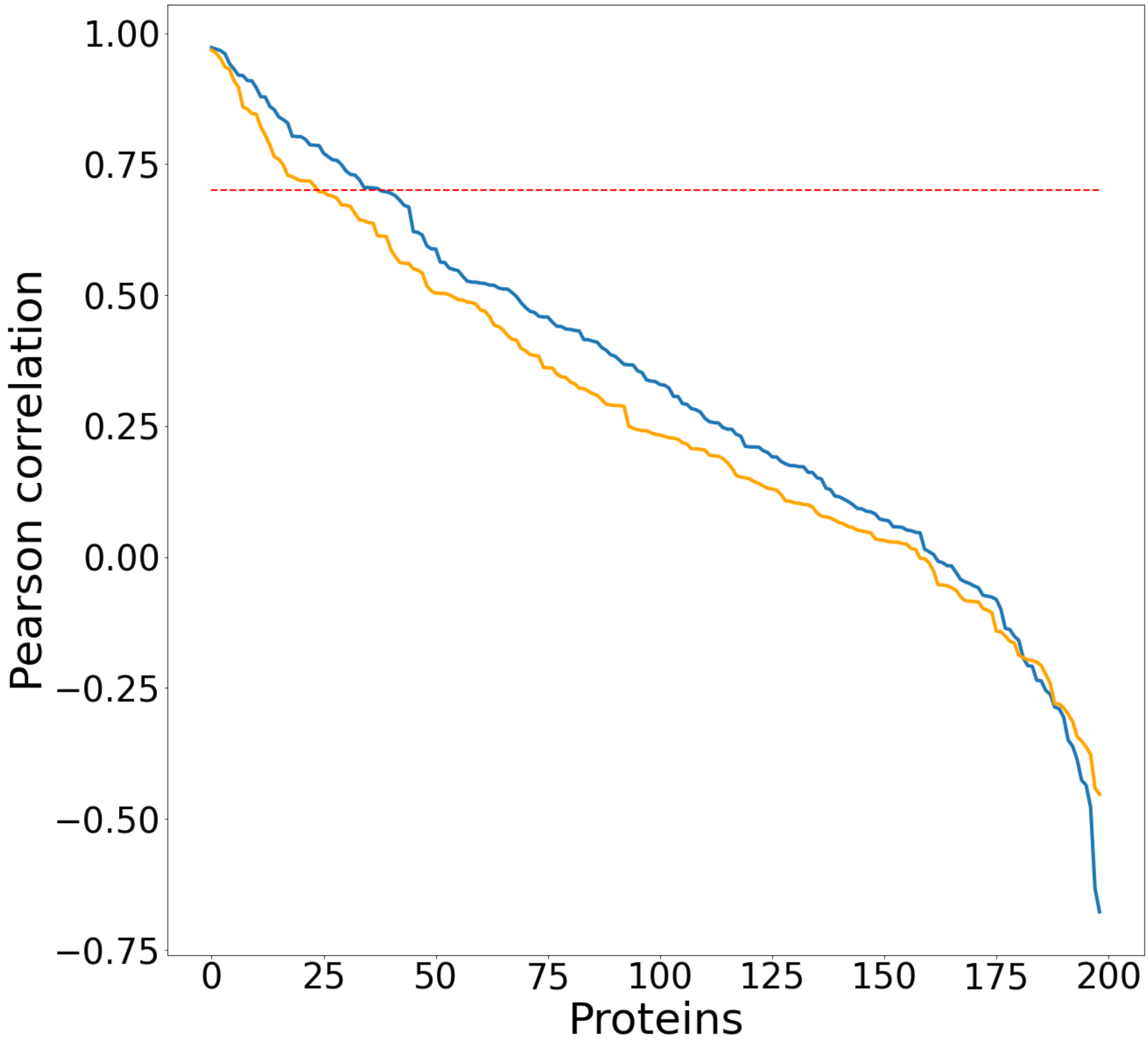

Figure S4. Pearson correlation of concentrations measured by both quantitative workflows for each protein in the full set of samples (orange) and in the set excluding the P1F2 sample (blue). Log2-normalized TPA concentration values were used for calculation. Dotted red line is displayed at the Pearson correlation value of 0.7.

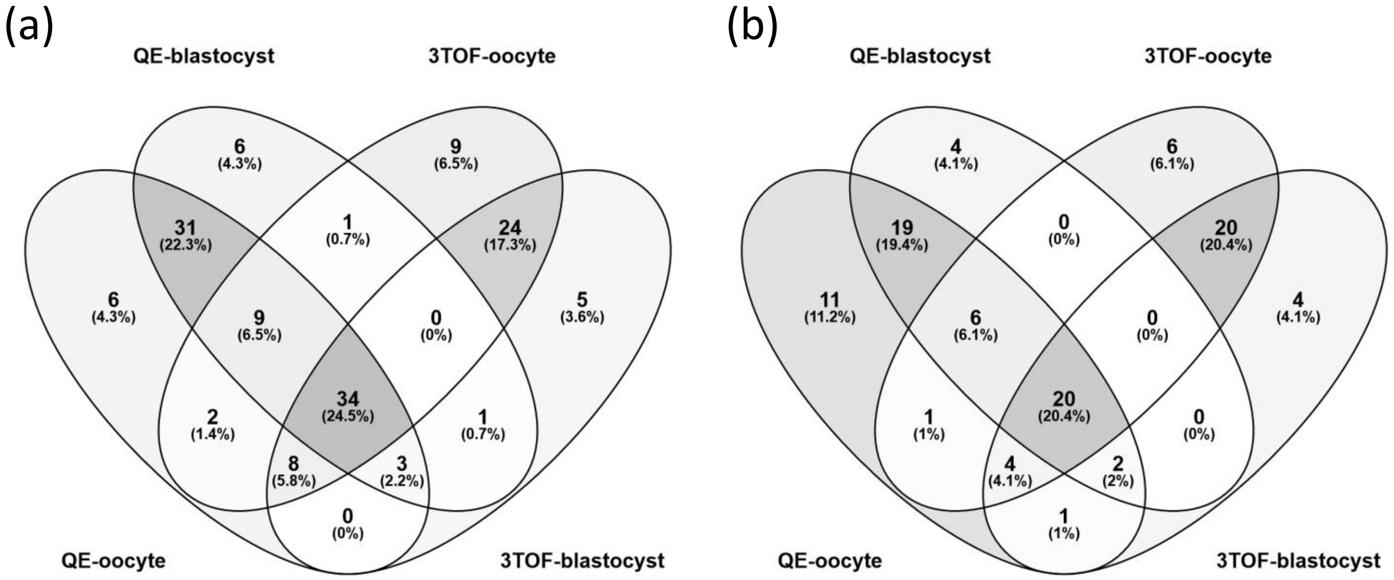

Figure S5. Proteins determined as statistically significant by two-factor ANOVA for the factor relating to inter-patient differences in each comparison (patient-oocyte status and patient-blastocyst status) for both quantitative workflows: **(a)** at 5% FDR, **(b)** at 1 % FDR.

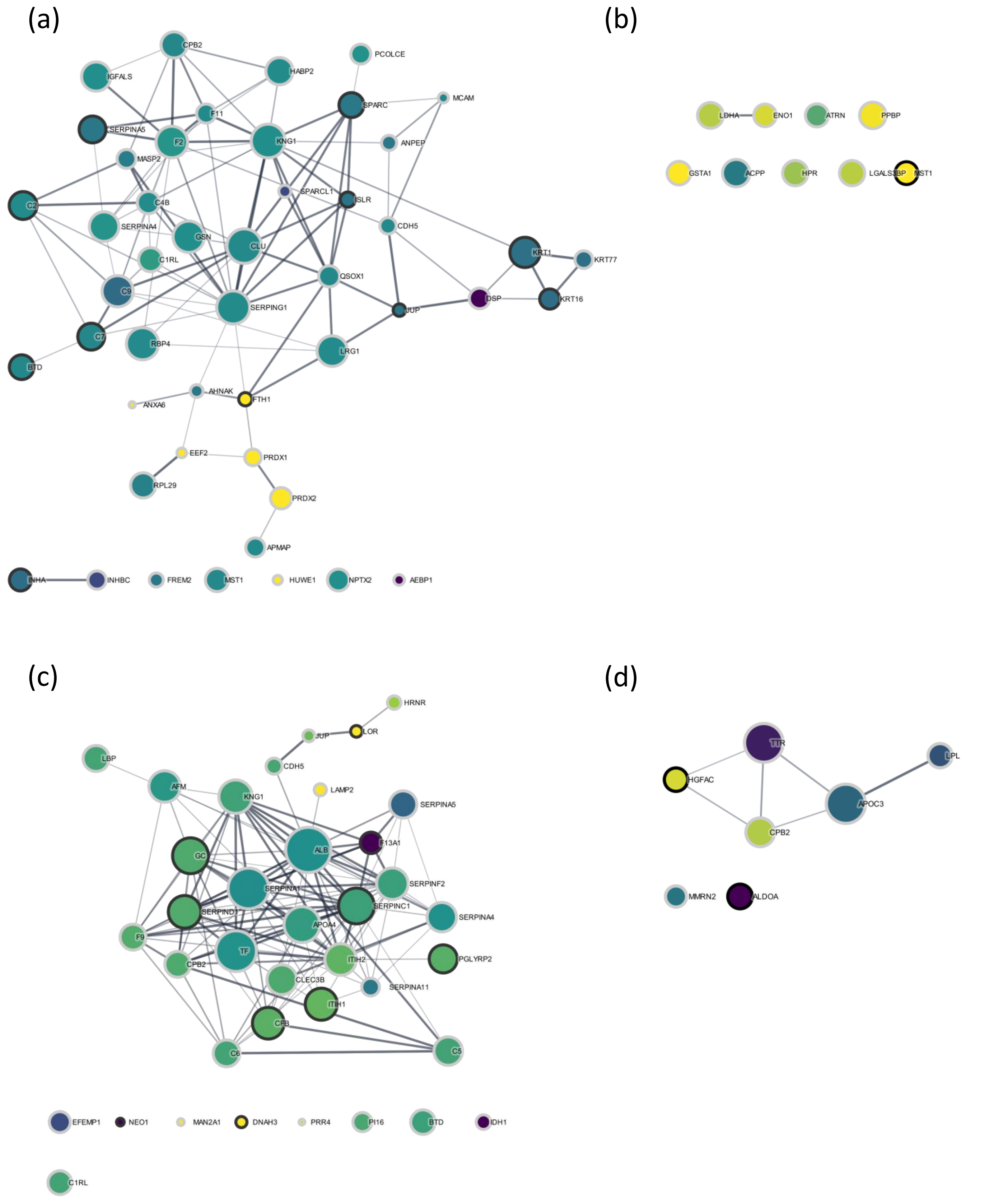

Figure S6. Interaction networks of all proteins determined as statistically significant by two-factor ANOVA for the factor relating to **(a,b)** oocyte maturity or **(c,d)** blastocyst development status for **(a,c)** Quad-Orbitrap or **(b,d)** Triple Quad-TOF at 5% FDR. Fill color relates to the fold change from 0.5 and below (yellow) through 1 (aquamarine) to 2 and above (purple). Node size represents log10 median abundance TPA concentrations in the test group (either mature oocyte or developed blastocyst). Black edges mark proteins statistically significant at 1% FDR.
